# Cocktails of Mycotoxins, Phytoestrogens, and Other Secondary Metabolites in Diets of Dairy Cows in Austria: Inferences from Diet Composition and Geo-Climatic Factors

**DOI:** 10.3390/toxins14070493

**Published:** 2022-07-15

**Authors:** Felipe Penagos-Tabares, Ratchaneewan Khiaosa-ard, Marlene Schmidt, Eva-Maria Bartl, Johanna Kehrer, Veronika Nagl, Johannes Faas, Michael Sulyok, Rudolf Krska, Qendrim Zebeli

**Affiliations:** 1 Institute of Animal Nutrition and Functional Plant Compounds, University of Veterinary Medicine Vienna, Veterinaerplatz 1, 1210 Vienna, Austria; felipe.penagostabares@vetmeduni.ac.at (F.P.-T.); 11774475@students.vetmeduni.ac.at (M.S.); 1545118@students.vetmeduni.ac.at (E.-M.B.); 11720032@students.vetmeduni.ac.at (J.K.); qendrim.zebeli@vetmeduni.ac.at (Q.Z.); 2 DSM—BIOMIN Research Center, Technopark 1, 3430 Tulln, Austria; veronika.nagl@dsm.com (V.N.); johannes.faas@dsm.com (J.F.); 3 Department of Agrobiotechnology (IFA-Tulln), Institute of Bioanalytics and Agro-Metabolomics, University of Natural Resources and Life Sciences Vienna, Konrad Lorenz-Strasse 20, 3430 Tulln, Austria; michael.sulyok@boku.ac.at (M.S.); rudolf.krska@boku.ac.at (R.K.); 4 Institute for Global Food Security, School of Biological Sciences, University Road, Belfast BT7 1NN, UK; 5 Christian-Doppler-Laboratory for Innovative Gut Health Concepts in Livestock (CDL-LiveGUT), Department for Farm Animals and Veterinary Public Health, University of Veterinary Medicine Vienna, Veterinaerplatz 1, 1210 Vienna, Austria

**Keywords:** mycotoxin, phytoestrogen, ergot alkaloid, co-exposure, dairy farming, feed safety

## Abstract

Dairy production is a pivotal economic sector of Austrian and European agriculture. Dietary toxins and endocrine disruptors of natural origin such as mycotoxins and phytoestrogens can affect animal health, reproduction, and productivity. This study characterized the profile of a wide spectrum of fungal, plant, and unspecific secondary metabolites, including regulated, emerging, and modified mycotoxins, phytoestrogens, and cyanogenic glucosides, in complete diets of lactating cows from 100 Austrian dairy farms. To achieve this, a validated multi-metabolite liquid chromatography/electrospray ionization–tandem mass spectrometric (LC/ESI–MS/MS) method was employed, detecting 155 of >800 tested metabolites. Additionally, the most influential dietary and geo-climatic factors related to the dietary mycotoxin contamination of Austrian dairy cattle were recognized. We evidenced that the diets of Austrian dairy cows presented ubiquitous contamination with mixtures of mycotoxins and phytoestrogens. Metabolites derived from *Fusarium* spp. presented the highest concentrations, were the most recurrent, and had the highest diversity among the detected fungal compounds. Zearalenone, deoxynivalenol, and fumonisin B1 were the most frequently occurring mycotoxins considered in the EU legislation, with detection frequencies >70%. Among the investigated dietary factors, inclusion of maize silage (MS) and straw in the diets was the most influential factor in contamination with *Fusarium*-derived and other fungal toxins and metabolites, and temperature was the most influential among the geo-climatic factors.

## 1. Introduction

Dairy production is the most important agricultural sector in the Republic of Austria, representing 18% of the national agricultural production [1]. Animal feeding is a fundamental element of milk production, affecting the rest of the productive chain, including aspects such as animal health and performance as well the quality and safety of the derived foods [2]. The composition of dairy cattle diets varies widely among farms and production systems worldwide, incorporating a broad range of ingredients including roughage, cereal grains, and agroindustrial by-products [2]. The physiological nature of ruminants makes forages (including pastures and conserved forages: silages, hay, and straw) the most adequate and important feed sources for dairy cattle [3]. Additionally, the incorporation of high-density energy dietary sources (concentrate feeds) is essential to achieve the high milk yields demanded and expected in modern dairy farming [2,4,5]. Such diversity of ingredients contributes to the dietary exposure to a broad spectrum of toxic, potentially toxic, and endocrine-disrupting fungal and plant secondary metabolites [6,7,8,9,10].

Crops and feedstuffs are susceptible to mould infection and colonization with subsequent contamination with mycotoxins and other fungal secondary metabolites during the feed-production chain, both pre- and post-harvest, influenced by several biotic and abiotic factors [11]. Probably based on the paradigm that ruminants are less susceptible to the negative effects of fungal toxins [12], most studies concerning mycotoxins and animal feeds have focused on monogastric animals and their main dietary sources (cereal grains). However, a wide spectrum of fungal metabolites (several of them toxic and potentially toxic), primarily produced by *Fusarium*, *Alternaria*, *Aspergillus*, *Penicillium*, and other fungal species, has been found in cattle feed sources beyond cereal grains [6,13]. Some of the mycotoxins are included in the European legislation, which currently establishes a maximum limit for aflatoxin B1 (AFB1) and guidance values (GV) for zearalenone (ZEN), deoxynivalenol (DON), T-2 and HT-2 toxins, fumonisins B1 and B2 (FB1 and FB2), and ochratoxin A (OTA), and thus, their occurrences and levels in feeds and diets have received strong attention [14,15,16]. More recently, monitoring studies on contamination frequency and levels of ergot alkaloids (EAs) and emerging mycotoxins in animal feeds have been highly advocated [17,18,19,20,21,22,23]. Consequently, the characterization of the implicated mycotoxin mixtures needs to be performed with an innovative and holistic approach based on multi-metabolite analyses to achieve an optimal risk assessment [24]. Such multi-metabolite analytic approaches are relevant because, additionally to single negative effects, there are multiple toxicological interactions (such as addition, synergism, potentiation, and antagonism) among mixtures of mycotoxins and other metabolites, which could have implications for health and reproduction. These interactions require more investigation [25,26]. Beyond toxic fungal metabolites, the dairy cattle diet contains substantial levels of plant secondary metabolites, some of which may induce unfavourable impacts on the health and/or reproduction of livestock, such as pyrrolizidine alkaloids, cyanogenic glucosides (CGs), and phytoestrogens (PEs) [27]. Phytoestrogens can act as endocrine disruptors, impairing reproductive functions, generating temporal infertility, and potentially reducing the productive efficiency of dairy herds [9,28,29,30,31]. Interestingly, mycoestrogens (such as ZEN, alternariol (AOH), and their modified forms) and PEs (such as isoflavones) have synergistic effects [32,33,34], which must be considered in the context of a complete risk assessment on livestock reproductive performance [35,36,37,38]. Fungal and plant growth as well as concentrations of secondary metabolites in the dietary components and finally in the complete rations are influenced by multiple factors such as plant species/varieties, infecting/colonizing fungal species/varieties, climatic conditions, geography, parasitic/symbiotic interactions, use of pesticides, and other agricultural practices utilized [39,40,41,42,43,44,45,46]. The most influential factors favouring mycotoxin contamination and PE production of feedstuffs and diets of dairy cows should be studied. More data in this field would contribute to developing pre- and post-harvest preventive and management strategies to reduce exposure and optimize the health and productive performance of livestock farming [6,39,46].

Several studies have analysed the occurrence of some mycotoxins in different types of feed ingredients, including in pastures, cereals, and silages [6,7,22,42,47,48,49]. Research on the incidence of mycotoxins and other fungal secondary metabolites in complete diets (i.e., TMR) of cattle has been carried out during the last decade; however, it is still scarce [50,51,52,53,54,55,56]. Targeting the dietary levels of toxins and endocrine-disrupting metabolites is vital to assessing the risks for impacts on health, reproduction, and production [10,24]. Moreover, the whole-diet approach applied across many farms with different farm characteristics and feeding management could reveal true high-risk ingredients in dairy rations. Thus, the current study determined the frequency, levels, and co-occurrences of a wide spectrum of mycotoxins, PEs, and other secondary metabolites in representative samples of lactating cows’ diets in 100 Austrian dairy farms, using a validated multi-metabolite liquid chromatography/electrospray ionization–tandem mass spectrometric (LC/ESI–MS/MS) method. Inclusion levels of the basal feed ingredients and their characteristics (chemical composition, particle size, hygienic status), dietary forage proportion, and geo-climatic factors (such as altitude, temperature, relative air humidity, and rainfall) were evaluated for their contribution to the dietary concentrations of mycotoxins, PEs, and other secondary metabolites.

## 2. Results

### 2.1. Characteristics of the Diets

#### 2.1.1. Type of Rations and Main Dietary Components

The participating farms fed three kinds of dietary rations to cows: (i) partial mixed ration (87%) and (ii) exclusively forage-based mixed rations (11%), both with separately fed concentrate, as well as (iii) total mixed rations (2%). The frequency and rate of the inclusion levels of the main dietary ingredients in the rations of Austrian dairy cows are shown in Table 1. Grass silage (GS) and MS were the most common forages incorporated in the rations of the visited Austrian dairy farms, presenting frequencies of inclusion over 80% and representing maximums of around 87% and 59% of the rations, respectively. About 60% of the farms used straw in the rations, with maximal inclusion of 10% on a dry-matter basis. Hay was included in around 18% of the evaluated diets, representing from 0.6% to 30% of the ration. Wet brewery’s spent grains (BSG) were included in 27% of the diets, with the maximal inclusion level of 13.5% of the total diet. Other silages (e.g., wheat, oats, barley, sunflower, and beep pulp) were included in 10% of the diets, with a maximal inclusion of 23.6% of the rations. The average forage-to-concentrate ratio was 66:34 (Table 1).

#### 2.1.2. Chemical Composition and Particle Size Distribution of Basal Rations

Farms showed variation in the chemical (proximate) composition of the basal ration (Table 1). The dry matter of the basal rations ranged from 25.7% to 54.6%. The basal rations contained an average of around 50% neutral detergent fibre (NDF), ranging from 36.8% to 75.2%. Non-fibre carbohydrate (NFC) ranged from 0.4% to 41.3% (average: 23.3%), and crude protein ranged from 10% to 21.2% (average: 15.4%). Values of ash and crude fat also showed a wide range. Farms used rations with considerable variation in terms of the distribution of the particle sizes (Table 1). Large particles (>19 mm) represented the main particle size in the ration, accounting for 46.8 ± 18% (mean ± SD) of the ration (as-fed basis). Particles of 8–19 mm and 1.18–8 mm represented similar proportions in the ration, with averages of 22.7% and 25.6%, respectively. Finally, the proportion of fine particles (<1.18 mm) represented on average 4.6% of the ration. The value reached a maximum of 13.7% (Table 1).

#### 2.1.3. Hygienic Status of the Main Dietary Ingredients

The hygienic status of the main components of basal rations (GS, MS, straw, hay, BSG, and concentrate) was determined by sensory evaluation and scored as “proper”, “minor deficiencies”, “significant deficiencies”, and “vast deficiencies” according to Kamphues et al., 2014 [57]. Most samples (>80%) of dried feedstuffs including straw, hay, and concentrates showed a proper hygiene score (Table 1). Wet conserved feeds presented major hygienic status concerns. MS was the feedstuff most often (over 50%) detected for hygiene deficiencies (minor to vast deficiencies). Ensiled grass presented minor deficiencies in hygienic status in 30% of the samples, significant deficiencies in 8%, and vast deficiencies in 3%. Around 44% of the BSG was not in proper hygienic conditions.

#### 2.1.4. Geo-Climatic Factors

The climate conditions of the participating dairy farms were retrieved from the database of the Central Institution for Meteorology and Geodynamics of Austria and are shown in Table 1. Farms were in regions within altitudes ranging from 262 to 1300 m.a.s.l. The average temperature of the month of sampling (May 2019 to September 2020) ranged from −0.8 °C to 22.4 °C. The average temperature during maize’s growing season (June–September) varied between 13 °C and 22 °C, with an average of 18.7 °C. The relative air humidity during the maize’s growing season was on average 70.1%, fluctuating from 60.3% to 78%. The accumulated rainfall from June to September during the maize growing season was on average 294.5 mm, with minimum and maximum values of 178 mm to 594 mm, respectively (Table 1).

### 2.2. Occurrence and Concentrations of the Detected Metabolites

#### 2.2.1. Groups of Metabolites

**Figure 1:**
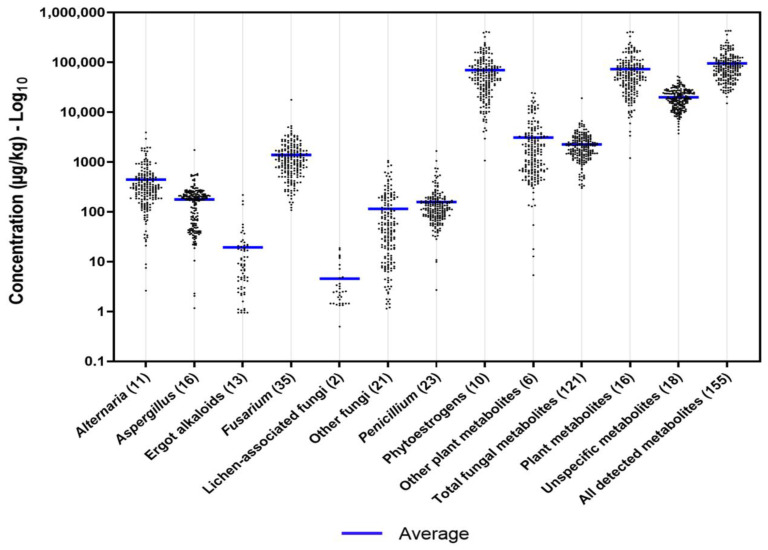
Scatter plot for concentrations (log_10_) of metabolite groups detected in whole diets of lactating cows (*n* = 198) from Austrian dairy farms. The total number of metabolites detected per group is shown in parentheses.

In total, 155 out of 863 targeted fungal, plant, and unspecific metabolites were detected in the analysed diets of lactating dairy cattle (Supplementary Table S1), consisting of 121 fungal compounds (including over 40 known mycotoxins), 17 plant metabolites, and 18 unspecific metabolites (Table 2). Their occurrences and respective average (with SD), median, and range of concentrations (expressed on a dry-matter basis in μg/kg) are indicated in Table 2. The detected metabolites were categorized in groups based on their main producers, consisting of *Alternaria*, *Aspergillus*, *Fusarium*, *Penicillium*, lichen-associated fungi, other fungal species, other plant metabolites, and unspecific (i.e., derived from fungi, bacterial and/or plants) metabolites, or corresponding to the kind of metabolites, such as EAs and PEs, according to previous reports [42,58,59]. Fusarial metabolites were detected in all samples and with the highest grade of diversity, with 35 different compounds identified (Table 2). Lower numbers of detected metabolites were derived from *Penicillium* (23), other fungal species (21), *Aspergillus* (16), *Alternaria* (11), and EAs (13). High occurrences (>90%) were detected for the groups of fungal metabolites (*Fusarium*, *Alternaria*, *Aspergillus*, and *Penicillium*), except for the total EAs (32.3% of total samples) and compounds produced by lichen-associated fungi (16.2%) (Table 2). Regarding the dietary contamination levels, the group of fungal metabolites with the highest average concentration was *Fusarium* (1380 μg/kg), followed by *Alternaria* (445 μg/kg), *Penicillium* (205 μg/kg), *Aspergillus* (177 μg/kg), other fungi (115 μg/kg), EAs (19.5 μg/kg), and minor grade lichen-associated fungi (4.57 μg/kg) (Table 2). As displayed in Figure 1, the distribution of the concentrations among groups of metabolites varied widely. 

As presented in Table 2, ten different PEs and six additional plant metabolites were identified across all samples. Most of these plant metabolites occurred in high frequencies and high concentrations, with average concentrations of total PEs and other plant metabolites above 70,000 μg/kg and 3000 μg/kg, respectively. A high degree of variation among the samples was marked (Figure 1), with ranges from 1080 μg/kg to 411,000 μg/kg for total PEs and from 5.37 μg/kg to 24,500 μg/kg for the total of other secondary plant metabolites (Table 2). All diets were detected for unspecific metabolites (Table 2). The total concentrations of this category presented an average of 20,000 μg/kg and ranged from 3740 μg/kg to 52,400 μg/kg. The concentration heterogeneity was evident for this group of metabolites (Figure 1).

#### 2.2.2. Mycotoxins Included in the EU Legislation and Related Compounds

**Figure 2:**
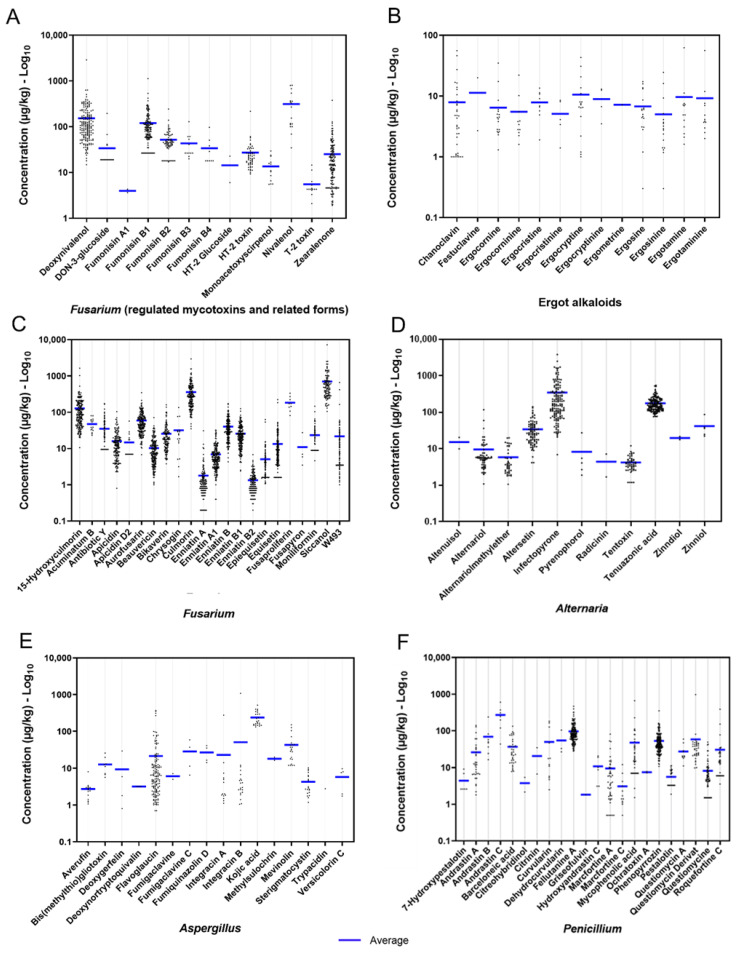
Scatter plots illustrating the distribution of individual concentrations (log_10_) of mycotoxins and fungal metabolites presented in complete diets of Austrian dairy cows. (**A**) *Fusarium* mycotoxins considered in the legislation, as are related compounds, (**B**) ergot alkaloids, (**C**) other mycotoxins and metabolites from *Fusarium*, and (**D**) mycotoxins and metabolites derived from *Alternaria*, (**E**) from *Aspergillus,* and (**F**) from *Penicillium*. The mean, SD, median, minimum, and maximum values are presented in Table 2.

The mycotoxins with GV in the European legislation but not the strongly regulated AFB1 were found in the dietary rations tested in the present study (Table 2). The level of occurrences and heterogeneity in concentrations across samples differed among these mycotoxins (Figure 2A). Accordingly, DON, ZEN, and FB1 were the most abundant and frequently found regulated mycotoxins (Table 2). Type A trichothecenes, T-2 toxin, and HT-2 toxin were detected in frequencies <30%. Metabolites structurally and toxicologically related to the regulated fusarial metabolites, including DON-3-glucoside, nivalenol (NIV), monoacetoxyscirpenol, HT-2 glucoside, FA1, FB3, and FB4, occurred in the studied diets but at lower frequency compared to their parental form (Table 2, Figure 2A). Of these, NIV showed the highest concentration (range: 34.6–804; mean 311 μg/kg). The mycotoxin OTA (produced mainly by *Penicillium* spp. but also by *Aspergillus* spp.) was detected only in 1% of the samples and in low concentrations (<8 μg/kg). In total, 13 different EAs were identified. The individual levels of EAs detected in the evaluated samples of diets’ averages were below 12 μg/kg and presented maximum concentrations less than 65 μg/kg, and their occurrences were lower than 20% (Table 2). The concentration distribution across samples was similar among the EAs (Figure 2B).

#### 2.2.3. Emerging Mycotoxins

This study detected 20 compounds classified as emerging toxins [60,61,62] (Table 2). Emerging mycotoxins were derived mainly from the genera *Fusarium* (15) and, to a lower degree, from *Alternaria* (3), *Aspergillus* (1), and *Penicillium* (1) (Table 2). In total, five forms of enniatins (ENNs) were detected, including ENN A, ENN A1, ENN B, ENN B1, and ENN B2. All of them occurred in at least 65% of the total samples. ENN B, ENN B1, and ENN A1 presented the most frequent detection. The average levels of the individual ENNs were ≤40.2 μg/kg, and the maximum levels were not superior to 180 μg/kg. The sum of ENNs presented an average of 75 μg/kg, ranging from 7.36 μg/kg to 324 μg/kg. Other frequently found metabolites (presented in more than 80% of analysed diets) were aurofusarin (AUR), beauvericin (BEA), bikaverin, culmorin, 15-hydroxyculomorin, epiequisetin, equisetin, and siccanol. Despite the high frequency of contamination with *Fusarium*-produced emerging mycotoxins in the samples, the mean and median concentrations remained below 400 μg/kg, except for siccanol (mean: 709 μg/kg; median: 494 μg/kg; range: 106 μg/kg–7220 μg/kg). All fusarial emerging mycotoxins showed noticeable variations among samples (Figure 2C). The emerging toxins and mycoestrogens derived from *Alternaria* were detected, consisting of AOH, alternariol methyl ether (AME), and tenuazonic acid (TeA). These metabolites were detected at rates between 40% and 80% of the samples, with average concentrations below 180 μg/kg. Among the *Alternaria* metabolites, TeA presented the highest frequency (78.8%) and contamination levels (range: 76.1 μg/kg–549 μg/kg) (Table 1), but its concentrations across samples were more homogenous than infectopyrone (Figure 2D). For *Aspergillus*-derived emerging mycotoxins, the carcinogenic and aflatoxin precursor sterigmatocystin (STC) was detected in 17.2% of the samples, with an average concentration of 3.6 μg/kg, ranging from 1.19 μg/kg to 10.3 μg/kg (Table 2). Mycophenolic acid (MPA) and roquefortine (ROQ) C were detected with frequencies around 20%, showing concentrations varying from 1.52 μg/kg to 661 μg/kg and from 3.56 μg/kg to 387 μg/kg, respectively.

#### 2.2.4. Other Mycotoxins and Metabolites from *Fusarium*, *Alternaria*, *Aspergillus*, and *Penicillium*

Additionally, many other less-known mycotoxins and metabolites associated with *Fusarium*, *Alternaria*, *Aspergillus*, and *Penicillium* were found in the diets of Austrian dairy cows (Table 2, Figure 2). Metabolites produced by *Fusarium*, including 15-hydroxyculmorin, antibiotic metabolites, and W493, were found in more than 40% of the samples, whereas acuminatum B, apicidin D2, chrysogine, fusaproliferin, and fusapyrone had lower occurrences at below 10%. Concerning other compounds derived from the genus *Alternaria*, infectopyrone (78.3%) and altersetin (47%) were the most frequently found metabolites, after the previously mentioned TeA (Table 2). In terms of concentrations, infectopyrone was the major contaminant produced by *Alternaria* (Figure 2D). Multiple compounds produced by members of the genus *Aspergillus* and *Penicillium* were detected in diverse frequencies of occurrence and contamination levels. Most of the *Aspergillus* and *Penicillium* secondary metabolites were detected in rates lower than 10% of the samples and presented average and median concentrations below 100 μg/kg. For *Aspergillus*-derived metabolites, while kojic acid showed the highest mean concentration (165 μg/kg), flavogluacin was the most frequently found metabolite and presented high concentration heterogeneity across samples (Table 2, Figure 2E). Fellutanine A and phenopyrrozin were the most frequently found *Penicillium* metabolites and had relatively high mean concentrations as compared to other *Penicillium* metabolites (Table 2, Figure 2F).

#### 2.2.5. Metabolites from Lichen-Associated Fungi and Other Fungi Genera

**Figure 3:**
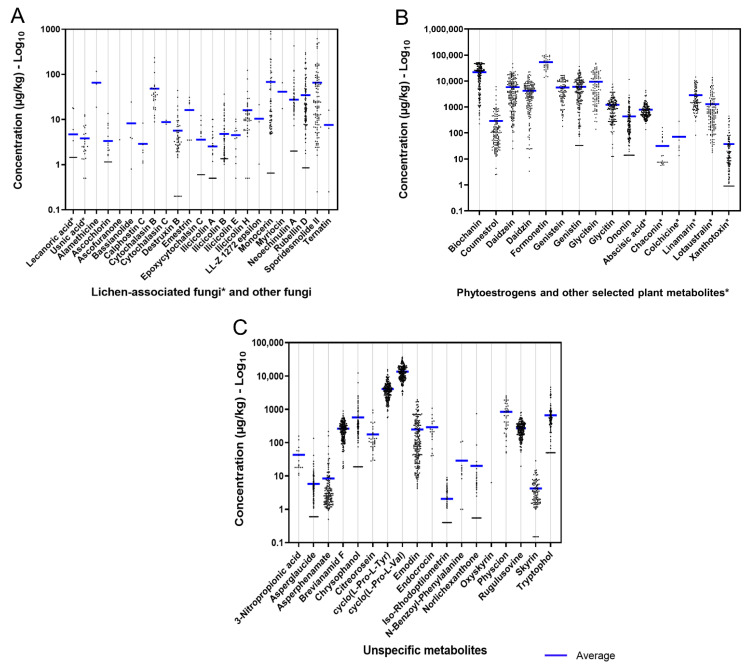
Scatter plots illustrating the distribution of individual concentrations (log_10_) of metabolites produced by (**A**) lichen-associate fungi and other fungal species, (**B**) phytoestrogens and other plant-derived metabolites, and (**C**) unspecific metabolites (produced by fungi, plants, and/or bacteria) presented in complete diets of Austrian dairy cows. The exact average, SD, median, minimum, and maximum values are presented in Table 2.

The occurrence of the individual metabolites produced by other fungal species was under 40%, with the exception of rubellin D (57.1%) and sporidesmolide II (51.5%) (Table 2). Monocerin was the most abundant compound in this group (average: 68.1 μg/kg; range: 0.65–893 μg/kg). The ilicicolins A, B, E, and H occurred in concentrations below 125 μg/kg. The two lichen-derived metabolites detected were usnic acid (11.6%, 0.50–12.7 μg/kg) and lecanoric acid (6%, range: 1.45–18.1 μg/kg). Despite relatively low concentrations, the concentrations of other fungi- and lichen-derived metabolites varied considerably among samples (Figure 3A).

#### 2.2.6. Plant Secondary Metabolites (Phytoestrogens and Other Plant Metabolites)

The detected PEs in the rations consisted of nine isoflavones, namely biochanin, daidzein, daidzin, formonetin (synonym: formononetin), genistein, genistin, glycitein, glycitin, and ononin, and a coumestan (coumestrol). With the exception of formonetin (21.2%), all of the phytoestrogens occurred in ≥70% of the samples (Table 2). The contamination levels of isoflavones biochanin, daidzein, daidzin, genistein, genistin, and glycitein were higher than 4500 μg/kg. The metabolites with the highest contamination levels found in this study were formonentin (average: 78,700 μg/kg; range: 13,800–289,000 μg/kg) and biochanin (average: 21,900 μg/kg; range: 226–52,100 μg/kg). Regarding other plant metabolites, abscisic acid, lotaustralin, and xanthotoxin occurred in more than 60% of the evaluated dairy cattle diets, whereas linamarin, chaconin, and colchicine presented lower occurrences (47%, 11%, 6%, and 4%, respectively). The cyanogenic glycosides linamarin (average: 2850 μg/kg; range: 82.5–14,200 μg/kg) and loustralin (average: 1300 μg/kg; range: 18.1–13,700 μg/kg) presented the highest levels within the category of other plant metabolites (Table 1, Figure 3B).

#### 2.2.7. Unspecific Metabolites (Derived from Multi-Kingdom Producers)

Unspecific metabolites can be produced by different and unrelated organisms belonging to diverse kingdoms (Plantae, Fungi, and/or Eubacteria). In this category, four metabolites, namely brevianamide F, cyclo (L-Pro-L-Tyr), cyclo (L-Pro-L-Val), and rugulusovine, were evidenced in all the assessed diets. The compounds asperglaucide, asperphenamate, chrysophanol, emodin, and tryptophol occurred at a rate superior to 50%. Skyrin, iso-rhodoptilometrin, citreorosein, norlichexanthone, and physcion were detected in frequencies between 15% and 50%. Low rates (<10%) of 3-nitropropionic acid, endocrocin, and N-benzoyl-phenylalanine were detected in the samples. The superior concentrations in the category of unspecific metabolites corresponded to the bioactive cyclic dipeptides cyclo (L-Pro-L-Val) (average: 13,700 μg/kg; max.: 36,900 μg/kg), and cyclo (L-Pro-L-Tyr) (average: 4100 μg/kg; max.: 15,400 μg/kg), as well as the alcohol tryptophol (average: 1030 μg/kg; max.: 6380 μg/kg). The other metabolites of this group presented average concentrations lower than 850 μg/kg (Table 1, Figure 3C).

### 2.3. Co-Occurrence of Mycotoxins, Phytoestrogens, and Other Secondary Metabolites

**Figure 4:**
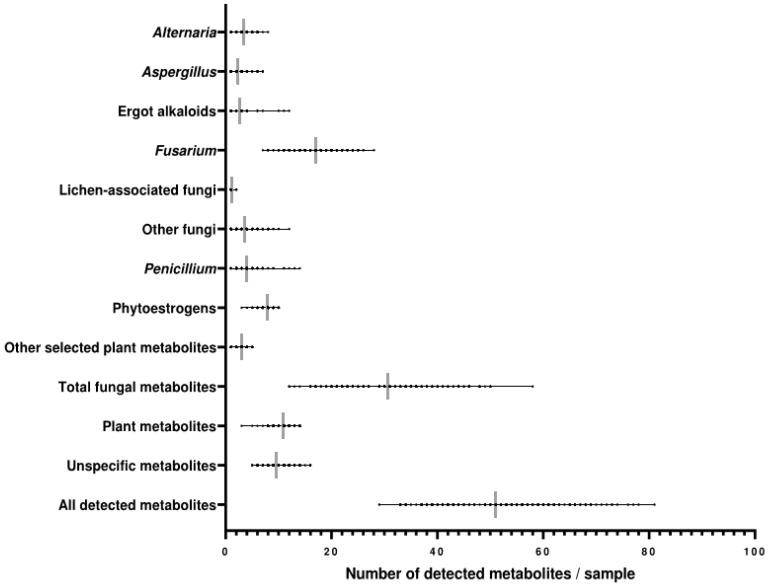
Scatter plots showing the number of metabolites per sample in each metabolite group detected in whole diets of lactating dairy cows in Austria. The grey lines indicate the average numbers of detected metabolites per sample.

Apparent differences in the number of detected metabolites per sample were observed (Figure 4). Samples were co-contaminated with 29 to 81 metabolites, with an average of 51 co-contaminating metabolites per sample. Considering metabolites derived from fungi, the number per sample ranged from 12 to 58, with an average of 31 compounds. On average, each sample presented a mixture of 8 PEs. The samples contained a mean of 11 plant-derived and 10 unspecific metabolites, ranging from 3 to 14 and from 5 to 16 metabolites per sample, respectively (Figure 4).

**Figure 5:**
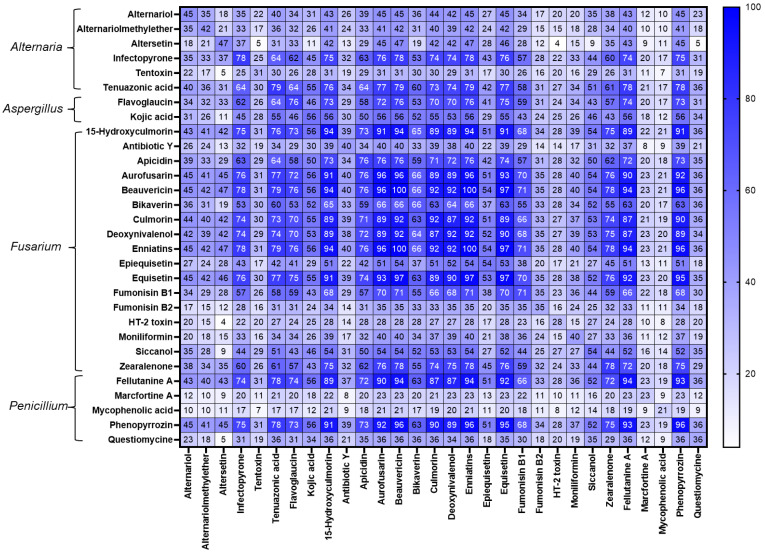
Heatmap indicating the co-occurrence (%) of the selected mycotoxins, which occurred in ≥20% of total samples, detected in the diets of Austrian dairy cows.

The frequencies of co-occurrence analyses between mycotoxins are presented in Figure 5. The most recurrent combinations of mycotoxins detected in the complete rations of dairy cows were between fusarial emerging mycotoxins (ENN A1, ENN B, ENN B1, 15-hidroxyculmorin, AUR, and equisetin) (100%), which presented co-occurrences over 90%. ENN A1 and ENN B (94%), ENN A1 and ENN B (94%), and ENN A1 and ENN B1 were widespread combinations. The combinations of the other *Fusarium* regulated mycotoxins ZEN and DON (75%), DON and FB1 (68%), and ZEN and FB1 (59%) were considerably frequent. *Aspergillus*-derived metabolites such as flavoglaucin and kojic acid presented co-occurrence with fusarial metabolites up to 79%. Remarkably, more than one-third of the samples showed co-contamination between several emerging *Fusarium* (ENNs, BEA, AUR) and *Alternaria* (AOH, AME, and TeA) mycotoxins.

**Figure 6:**
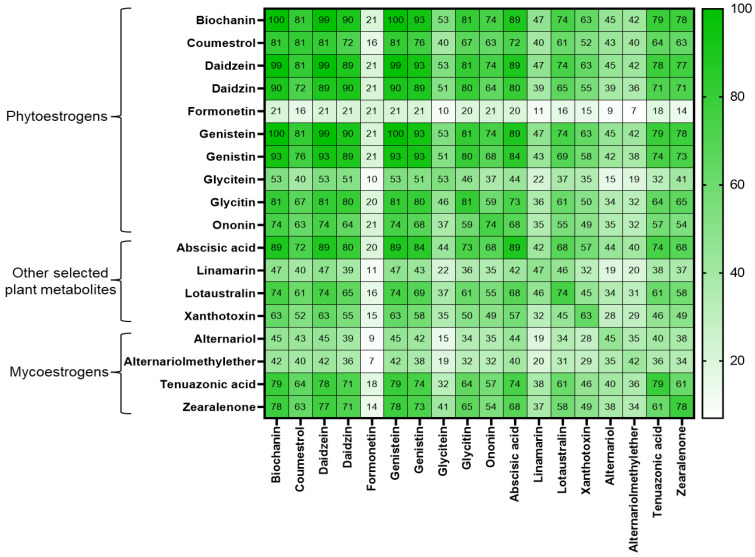
Heatmap indicating the co-occurrence (%) of phytoestrogens, other plant-derived metabolites, and mycoestrogens detected in the whole diets of Austrian dairy cows.

The co-occurrence rates of PEs, other plant-derived metabolites, and mycoestrogens (AOH, AME, TeA, and ZEN) are illustrated in Figure 6. All tested samples presented co-contamination between biochanin and genistein. Samples often presented with mixtures of PEs with high occurrences (>70%), including the metabolites coumestrol, daidzein, daidzin, genistein, and genistin. Many of the PEs co-occurred with the mycoestrogens in more than 30% of the samples. Particularly, ZEN and TeA showed relatively higher co-occurrences of PEs compared with the mycoestrogens from *Alternaria.*

### 2.4. Dietary Composition and Geo-Climatic Factors in Relation to the Concentration of Mycotoxins, Phytoestrogens, and Other Secondary Metabolites

Correlations between recorded dietary and geo-climatic factors (see Table 1) with the contamination with fungal (toxic) metabolites of interest were screened using Spearman correlation analysis. Based on this approach, we observed some potential factors (i.e., Spearman rank correlation coefficient (ρ) ≥ 0.3). Among dietary ingredients, we found that MS showed the highest correlations with the concentration of *Fusarium* mycotoxins such as DON (ρ = 0.40, *p* < 0.001), sum of type-B trichothecenes (ρ = 0.38, *p* < 0.001), ZEN (ρ = 0.30, *p* < 0.001), CUL (ρ = 0.32, *p* < 0.001), BEA (ρ = 0.42, *p* < 0.001), and total *Fusarium* metabolites (ρ = 0.36, *p* < 0.001). The content of straw in the ration showed a significant positive correlation with infectopyrone (ρ = 0.62, *p* < 0.001), total *Alternaria*-derived metabolites (r = 0.52, *p* < 0.001), and total fungal metabolites (ρ = 0.33, *p*= < 0.001). The dietary proportion of BSG presented a significant positive correlation with the contamination levels of many *Fusarium*-derived mycotoxins such as ENN A1 (ρ = 0.47, *p* < 0.001), ENN B1 (ρ = 0.38, *p* < 0.001), and total ENNs (ρ = 0.35, *p*< 0.001). The proportion of feed particles with size between 1.18 and 8 mm presented a low positive correlation with the presence of fusarial metabolites (ρ = 0.33, *p* < 0.001), whereas the proportion of ration with a size longer than 19 mm correlated negatively (ρ = −0.33, *p* < 0.001). Of the geo-climatic conditions studied (see Table 1), the temperature during the maize’s growing season showed a positive correlation with type B trichothecenes (ρ = 0.36, *p* < 0.001), AUR (ρ = 0.33, *p* < 0.001), BEA (ρ = 0.37, *p* < 0.001), and total fusarial metabolites (ρ = 0.30, *p* < 0.001).

**Figure 7:**
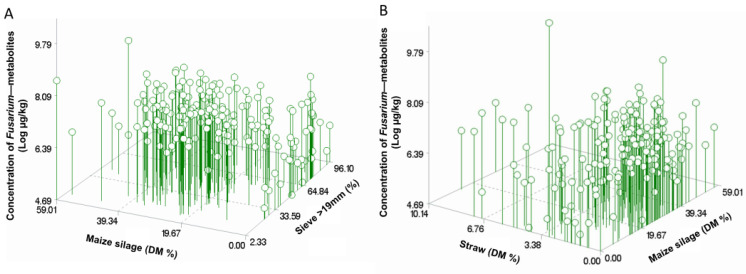
Scatter plots in 3D of the combined influence of dietary factors on the levels of *Fusarium* metabolites (Log-µg/kg) in diets of dairy cows in Austria. (**A**) Influence of content (% DM) of maize silage and particle size > 19 mm (%). (**B**) Influence of the content (% DM) of maize silage and straw.

Multiple regression models of the log-transformed concentration values of compounds derived from species of *Alternaria*, *Fusarium*, and *Penicillium*, total fungal and some individual mycotoxins such as ZEN, DON, ENNs, BEA, CUL, as well as the sum of FB1 and FB2 are presented in Table 3. Influences of some dietary factors based on the simple correlation method were confirmed by a multiple regression approach. Importantly, the multiple regression approach revealed a joint effect of multiple factors attributed to the dietary concentration of mycotoxins. Inclusion levels of MS and straw, the proportion of particles >19 mm, and dietary NFC content affected total concentrations of *Fusarium* metabolites. Together, these factors explained 52% of the variance, which is the highest value observed in this present study. Specifically, the proportion of MS and its combination with straw positively influenced the contamination levels of *Fusarium*-derived metabolites (slope = 0.004, *p* = 0.042, Table 3 and Figure S1). As shown in Figure 7, at the same level of MS, farms using more straw showed higher *Fusarium* contamination and vice versa. Interestingly, a quadratic effect of the proportion of MS was observed, and the total *Fusarium* metabolite peaked at an MS level around 30–35% of the basal diet DM before dropping to a higher MS level (Figure 7). A similar outcome was observed via logistic regression analysis that estimated an odds ratio of 1.05 (95% confidence limits: 1.01–1.08) and predicted a close to 75% chance for high loads of *Fusarium* metabolites at MS inclusion level of 30% of the diet DM (Supplementary Data Table S2 and Figure S1). For individual fusarial mycotoxins, the inclusion of MS positively influenced the contamination level of DON and emerging mycotoxins BEA, CUL, and SIC, while the proportion of particles >19 mm negatively influenced the contamination of ZEN, CUL, SIC, and total *Fusarium* metabolites. However, this depended on the inclusion level of MS. As shown in Figure 7, when no MS was used, the contamination of *Fusarium* metabolites increased with an increment in the proportion of particles >19 mm. With the inclusion of MS, the effect of MS dominated the effect of the proportion of particles >19 mm. Only ZEN was related to the level of ether extract (i.e., crude fat) of basal diet and hygiene score of GS. In agreement with simple correlation analysis, the inclusion level of straw affected the contamination level from *Alternaria* but with a significant quadratic effect. The influence of the temperature during the maize’s growing season was only confirmed for the BEA contamination. The influence of the dietary proportion of BSG on the total ENN commination was confirmed by the multiple regression approach; however, it explained only 19% of the variance. In addition to the factors listed above, the ash content in basal diets (with a positive quadratic response) contributed to the concentration of total fungal metabolites. None of the factors studied substantially explained the concentration of phytoestrogens and plant metabolites (data not shown).

## 3. Discussion

*Fusarium* metabolites were the most relevant fungal contaminants in the rations of dairy cattle surveyed in the present study, corroborating again the importance of *Fusarium* as one of the most widespread mycotoxigenic species in crops and the main contributor to mycotoxin contamination in animal feeds [7,64,65]. Among the EU-regulated mycotoxins, the type B trichothecene DON (occurrence: 92%) was predominant, followed by the mycoestrogen ZEN (77%) and FB1 (71%). The type A trichothecenes, T-2 and HT-2 toxin, which are more cytotoxic than the type B trichothecenes [66], were detected in low frequencies (12% and 18%, respectively) and concentrations (on average < 30 µg/kg). The contamination levels of these regulated mycotoxins were not over the guidance values of the European Union for feeds of dairy cattle. However, it has been proven that even dietary contamination under the EU values can negatively affect the performance, digestion, and immunity of dairy as well as beef cattle [67]. We showed that numerous non-regulated emerging and modified toxins produced by *Fusarium* spp. were even more recurrent and presented higher contamination levels. Although the tangible implications resulting from exposure to modified and emerging mycotoxins are not properly characterized, it is known that these compounds interact with other well-recognised fungal toxins, increasing their toxicological activity [24,25]. The high occurrences, concentrations, and diversity of metabolites derived from *Fusarium* spp. confirm the omnipresence and relevance of this genus in the mycotoxin contamination of crops and animal feeds [64,65]. Emerging *Fusarium* mycotoxins ENNs and BEA have antibacterial and cytotoxic properties; however, their implications for health and performance in ruminants are underexplored [21,68]. Research on the impact of such kinds of compounds on rumen ecology and functionality is crucial [12,61,69]. Maize silage and straw were the main forage components that drove the increasing concentration of *Fusarium* metabolites, which lined up with previous studies in the Netherlands [70,71] and Spain [50]. Viable *Fusarium* spp. is rarely isolated in ensiled maize, suggesting that *Fusarium* species do not grow properly during the ensiling process [72]. However, it has been widely proposed that mycotoxins of *Fusarium* spp. are mainly produced during crop growing [73,74] and, therefore, field conditions such as temperature influence mould proliferation and mycotoxin synthesis, supporting the notion that global warming promotes mycotoxin contamination in crops and feeds [46,75,76,77]. Various studies mark the key effects of temperature and humidity on mycotoxin contamination [39,42,46]. Based on our correlation analysis, environmental temperature increments during the crop’s late growing season (June to September) and the sampling month were associated with a higher accumulated concentration of some *Fusarium* mycotoxins (type B trichothecens, AUR, and BEA) and *Penicillium* metabolites, respectively. However, its significance was not confirmed by the multiple regression approach, except for BEA. We also did not observe significance for humidity. This might be explained by the accuracy of available climatic data when studying dietary contaminations coming from multiple sources (self-produced and purchased feed as well as different time of storage). This means that spot or average climatic data do not match the concentration at sampling as precisely as studies of single feed sources such as pasture [42].

In general, we did not observe dietary concentrations of regulated mycotoxins exceeding the EU maximum limit and GVs. Compared to the earlier study in Spain by Rodriguez-Blanco et al. (2020), we observed higher occurrences of regulated *Fusarium* mycotoxins. The researchers studied a similar number of total mixed rations (*n* = 193) from different areas of Spain during the period from February 2016 to January 2018 and found that DON (16.6%), ZEN (16.0%), and the sum of FB1 and FB2 (34.2%) presented lower occurrences and slightly higher average concentrations than those found in our study. However, all the samples showed values under the EU recommendations [78]. Other mycotoxin surveys performed in several European countries have also evidenced high occurrences and contamination levels of *Fusarium* mycotoxin in MS [61,79]. Dreihuis et al. (2008) estimated the dietary intake of four mycotoxins (DON, ZEN, ROQC, and MPA) of high-producing dairy cows in different regions of the Netherlands. The detected mean concentrations of DON, ZEN, ROQC, and MPA in complete diets were 273 μg/kg, 28 μg/kg, 114 μg/kg, and 54 μg/kg, respectively. Consistent with our findings, they reported that MS was the major feed source of these mycotoxins in the diet [70]. Similarly, other studies underline MS as the potential feed source of *Fusarium* mycotoxins [61,78]. Matching our results, *Fusarium*-derived mycotoxins were the most recurrent fungal contaminants with the highest concentrations detected in total mixed rations of Brazilian feedlots [54]. Europe-based studies, including the present research, rarely report the detection of AFB1. Nevertheless, this was the case in a recent study on Lithuanian dairy farms [56]. In that study, the analysis of total mixed rations (*n* = 51) collected in 2019–2020 showed that 60.8% of the rations were positive for AFB1, 54.9% for DON, 49% for ZEN, and 29.4% for T-2 toxin, and AFB1 exceeded the maximum concentration limits in haylage samples [56]. Moreover, the maximum average concentrations of AFB1 and T-2 toxin were found in the GS samples, while some samples of ensiled maize had ZEN and DON concentrations exceeding the EU GVs. Relating to toxic compounds produced by Aspergilli, the absence of strongly regulated AFB1 and other AFs was expected, because the occurrence of these mycotoxins in central Europe has been considered rare [46]. However, we detected precursors of AFs, such as averufin, STC, and versicolorin C [79,80], albeit at low frequencies (<20%) and concentrations (<11 µg/kg). Regarding STC, it has been suggested that this mycotoxin can be produced pre-and post-harvest [81]. Like AFs, STC is a known carcinogenic with immunotoxic and immunomodulatory activity. In general, the information available on exposure data of dairy cows to these precursors of AF is still very limited [18,60]. Fungi of the genus *Bipolaris*, *Chaetomium*, and *Emiricella* are able to synthesize STC [82]. OTA, considered in the European regulation, is produced by *Penicillium* and *Aspergillus* spp. and presented very low occurrence and contamination levels in the present survey, which suggests that this mycotoxin presents a minor risk for Austrian dairy herds. Additionally, kojic acid, produced primarily by *Aspergillus* spp. but also by *Penicillium* and *Acetobacter* fungi [83], has been shown to have low toxicity for human macrophages, along with antibacterial and immunomodulatory properties [84,85,86]. In the present study, due to low frequencies as well as high heterogeneity of the metabolite composition among farms, we did not identify factors associated with the contamination of *Aspergillus* metabolites.

Other potentially harmful contaminants occurring in dairy cows’ diets were compounds derived from the genus *Alternaria*, some of which are considered emerging mycotoxins, such as AOH, AME, and TeA. Our study indicates that they are commonly presented in the diets of Austrian dairy cows. *Alternaria* spp. can grow and produce toxins in various crops in the field and post-harvest stage, causing considerable losses due to decomposition [87,88]. Our analysis further indicates that straw contributes to contamination from *Alternaria.* Data and information regarding occurrence in the feeds and toxicological implications of *Alternaria* toxins for livestock systems are still missing [88,89,90]. Our survey suggests that the occurrence of metabolites of *Alternaria* should not be ignored. For instance, TeA was the most frequently detected *Alternaria* metabolite in the diets of Austrian dairy cows. This mycoestrogen targets protein synthesis inhibition at the ribosomal level and is considered, concerning toxicity, the most important metabolite produced by *Alternaria* spp. [91]. The benzopyrene derivatives AOH and AME are not related to acute toxicity but are known for their genotoxic effects [92,93,94]. Moreover, AME, AOH, and TeA are also classified as mycoestrogens, showing strong synergistic estrogenic effects in combination with mycoestrogen ZEN even at very low concentrations [32,33,95]. Our co-occurrence analysis showed that 30% to 60% of the samples displayed co-contamination between ZEN and *Alternaria*-derived AOH, AME, and TeA.

The analysed diets presented several *Penicillium*-derived toxins, which are considered the most relevant post-harvest mycotoxins contained in silages [6,96,97,98,99,100]. However, the production of such toxins is also possible in the field [72,101]. MPA and ROQs are considered the most investigated *Penicillium* metabolites occurring in silage [6]. A common feature of many *Penicillium*-derived exometabolites such as MPA, ROQs, CIT, and OTA is their immunotoxic properties [102,103], which could interfere with the activity of innate and adaptative immune responses, predisposing the animals to secondary infectious diseases [104]. *Penicillium* toxins have been linked with appetite reduction, affecting nutrient efficiency, and increasing the incidence of abomasal ulcers, laminitis, gastroenteritis, abortion, and paralysis [105]. Additionally, toxins produced by *Penicillium* spp. Such as ROQ C have neurotoxic activity [106]. Despite their abundance in feeds and their potential harmful properties, the economic relevance of *Penicillium* mycotoxins in livestock farming is considered underestimated, because even though mycotoxins are believed to be rapidly metabolized by gut microbiota and hepatic enzymes [104,107,108,109], the detoxification process of mycotoxins can still be disrupted by their antimicrobial and hepatotoxic properties [104,107,110,111,112,113,114]. *Penicillium*-derived mycotoxins are mostly associated with storage, being detected frequently in mouldy spots of silages [100,115,116]. Although the temperature of the samplings’ month presented a negligible correlation (ρ = 0.20, *p* = 0.004) in our study, several studies performed under controlled conditions have proven that *Penicillium* growth and toxin production were strongly increased by higher temperatures [117,118,119,120]. *Penicillium roqueforti* has been described as the most predominant fungi in mouldy sections of silages in Austrian dairy farms [100]. Contamination with storage mycotoxins (mainly associated with *Penicillium*) can occur even in good-quality silages, since aerobic spoilage is practically unavoidable during feed-out [121]. Our findings did not reveal relationships between the hygienic status of the main feedstuffs (GS, MS, straw, hay, BSG, and concentrate) and the contamination levels, which has been reported previously in forages [122]. This can be explained by the fact that toxin production by a fungus does not correlate directly with its growth [123]. Over 30% of the evaluated diets contained EAs, toxic compounds associated with diverse endocrine, vascular, and neurological effects [124]. These can be commonly detected in cereal grains as well as in pastures [42,125,126]. Dietary exposure to EAs in dairy cattle can produce unspecific effects such as reduced productive and reproductive performance and acute clinical signs of ergotism including hyperthermia, convulsions, gangrene in distal portions of the body, and fatalities [127,128,129]. It was stated that feeds exceeding 250 µg/kg of EAs should not be fed to pregnant or lactating animals, because it could increase the risk of abortion and agalactia syndrome [126]. Additionally, further less-known metabolites are produced by other fungi detected in the diets of dairy cows. Some of them have antibacterial activity, for example, the anthraquinone rubellin D [130,131], illicicolins [132], monocerin [133,134], and cytochalasins [135,136].

Interestingly, the recent analysis indicates that as compared with contamination from other fungal groups, contamination of *Fusarium* metabolites can be explained to a greater extent by dietary factors that are mainly related to forage components. We demonstrated a complex relationship between MS, straw, and proportions of NFC and large particles (>19 mm) that drives the contamination of *Fusarium* metabolites in dairy cow diets. With our multiple regression approach, the independent factors can explain 50% of the variance, substantially higher than a previous study that used a simple correlation analysis [137]. As explained before, other studies have underlined MS as a potential feed source of *Fusarium* mycotoxins. This could be explained by the fact that starch induces mycotoxin production (e.g., trichothecenes) in *F. graminearum* [137]. Thus, the superior content of non-fibre carbohydrates (such as starch) in maize and cereal plants compared to other forages such as GS and hay could explain the elevated levels of mycotoxins and other secondary metabolites. Furthermore, we found that, in addition to MS, straw was likewise an influential forage component. Straw is often added to dairy cow diets containing high grains and high MS to compensate for physical characteristics (long fibre) of the diet. As minor dietary components, the hygienic as well as chemical characteristics of straw likely receive less attention as compared to main forage sources such as MS, GS, and hay. Mould infection could be present in straw but might not be screened out before feeding. We found that, in addition to *Fusarium* metabolites, straw was also a determinant for contamination with *Alternaria* metabolites. The black mould genus *Alternaria* includes various saprophytic, endophytic, and pathogenic species, which occur worldwide in different habitats such as soil, as well as on dead or dying plant tissues such as straw [138]. A recent Swiss survey targeting a broad spectrum of mycotoxins in barley products found higher concentrations of total fungal metabolites in straw than in grains [139]. Interaction of dietary large particle size with MS and with straw partly represented shifts in the physical characteristics of the diet based on the combination of forage choices. Dietary ash content did not influence concentrations of metabolites from *Fusarium, Alternaria*, or *Penicillium*, but it did influence total fungal metabolites. Its positive quadratic effect indicates that high fungal metabolite loads are associated with high dietary ash content. High dietary ash contents are an indicator of contamination with soil, which affects the hygienic quality of the feedstuffs. All in all, although the current data could prove partial roles of the main dietary factors, the outcome underlines that there is no single factor that dominantly influences the dietary contamination. Rather, the dominant influence comes through the combination of forage choice, management (particle length), and the hygienic status of feed sources.

Another novel outcome of the present study was related to PEs, which constitute the extensively recurrent class of metabolites contained in dairy rations. PEs are of concern in veterinary medicine and public health due to their endocrine-disrupting activity. These substances especially affect the reproductive organs and process, inducing infertility in livestock [140,141]. These metabolites are found primarily in *Leguminosae* plants, such as soy, but also in clovers (*Trifolium* spp.) and alfalfa/lucerne (*Medicago sativa*) [9,28,142]. Coumetrans such as coumestrol seem to be more potent in estrogenic activity [9,31]. The levels of coumestrol detected in diets of Austrian dairy herds in the present study were below the reported critical range (18–180 mg/kg) [141]. Their interaction with other estrogenic substances (such as mycoestrogens) is currently the focus of interest [38]. Other plant-derived compounds such as the cyanogenic glucosides linamarin and lotaustralin observed in the present study did not exceed the maximum limit (50 mg/kg) of total cyanogenic compounds established by the European Union [143]. Both compounds (linamarin and lotaustralin) have a relatively broad distribution in the plant kingdom, being found in high concentrations in cassava, soy, cereal, clovers, and other plant species [53,144]. In general, levels of these compounds in clover are not high enough to cause acute toxicity. Some clinical manifestations include dyspnoea, muscular contractions, and oedemas in mucous membranes [145]. Nevertheless, reports of cyanide poisoning of livestock are rare, suggesting that levels of cyanide- or HCN-producing compounds in the feed are generally low [143], as is also the case for the present study. The inclusion of hay showed a major correlation with both linamarin and lotastratin in this study. Among the unspecific metabolites detected were molecules of some biologically active toxins, which increase the toxicological complexity of the cocktails of secondary metabolites evidenced. These include, for instance, emodin (antibacterial and immunosuppressive) [146,147], 3-nitropropionic acid (neurotoxic) [148,149], skyrin [147], brevianamide F (cyclo-L-Trp-L-Pro) (antifungal and antibacterial) [150], cyclo (L-Pro-L-Tyr), and cyclo (L-Pro-L-Val) (antibacterial) [151,152]. The complex profiles of co-contamination with different mycotoxins, PEs, CGs, and other metabolites occurring in the diets of high-yielding dairy cows suggest unexplored and unpredictable synergistic as well as antagonistic toxic effects. Most of the detected metabolites represent unregulated compounds with a high diversity of biological and toxic activity, indicating that the characterization of the regulated contaminant in dairy feeds is only the tip of the iceberg of fungal and other environmental toxins.

## 4. Conclusions

This study underlined the omnipresence of a broad number of mycotoxins (most of them unregulated), Pes, and other metabolites occurring in diets of dairy cows in Austria. Overall, the Austrian dairy rations are safe when considering that the detected contamination levels were below the guidance values of the EU commission. Nevertheless, a vast majority of mycotoxins and metabolites are emerging ones, as well as less-known and less-studied fungal metabolites. Overall, *Fusarium*-produced metabolites and mycotoxins were the dominant fungal contaminants. Additionally, we found that dietary factors related to the use of forages, rather than concentrating sources, contribute to increased contamination of mycotoxins in Austrian dairy rations. Among typical forage sources, the content of MS and straw were the most influential factors linked to the concentration of *Fusarium* metabolites in the complete rations. The analysis further addressed the influences of characteristics of diets and hygienic substandard of forages. Individually, the detected mycotoxins represented a relatively low or safe level based on EU regulation and literature. However, the co-exposure to mycotoxins and other (fungal and plant) secondary metabolites has unpredictable effects. Our findings make clear that the evaluation of contamination with only regulated mycotoxins offers a limited picture of the possible toxicological risks to animal health, reproduction, and productivity. Therefore, it is crucial to elaborate surveillance and monitoring programs for a broad spectrum of metabolites in the dairy feed chain and to understand their toxicological effects. Furthermore, there is a need to increase awareness of the importance of feed management and nutrition as reduction and prevention measures for mycotoxin contamination in dairy production. Monitoring and further research based on multi-metabolite approaches in the dairy industry in other geographic regions are still necessary.

## 5. Materials and Methods

### 5.1. Sampling and Sample Preparation

**Figure 8:**
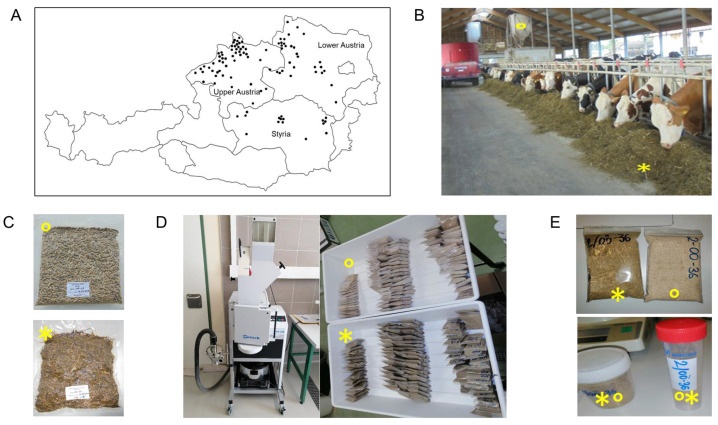
(**A**) representative sampling and sample preparation of whole diets of lactating dairy cows intended for multi-metabolite analysis via LC-MS/MS. (**A**) Map of locations of the selected dairy farms (*n* = 100) involved in this survey. (**B**) The representative sampling consisted of basal feed (total, partial, or forage mixed ration) collected from the feeding table (*) as well as samples of concentrated feeds (°). (**C**) Vacuum packing and preservation at −20 °C until sample preparation and subsequent analysis. Sampling preparation consisted of drying, (**D**) milling (to a particle size of ≤ 0.5 mm), and subsequent (**E**) pooling and homogenization according to the reported average intakes of basal feed and additional concentrate.

Under the agreement of written informed consent with the farmers, 100 dairy farms located in Lower Austria (*n* = 33), Upper Austria (*n* = 51), and Styria (*n* = 16), representing the 3 provinces leading the country’s dairy production, were involved in the survey, lasting from 2019–2020 (Figure 8A). The herd sizes (number of lactating cows) during both visits were on average 59 ± 15 SD lactating cows per farm, varying from 32 to 140 lactating cows per farm. Each representative sample of complete diets (*n* = 198) consisted of at least 30 incremental samples of mixed rations from the feeding table (feed bunk), and at least 30 subsamples of concentrate feed on the automatic feeders were collected. The final sample amount was 1–1.5 kg of each kind of sample (basal feed ration and additional concentrate) (Figure 8B). An additional sample of basal ration (approx. 1 kg) was collected for particle size determination. The samples were immediately vacuum-packed (−0.7 psi) and stored in the dark at −20 °C to avoid subsequent microbial spoilation until sampling preparation (Figure 8C). Sampling was performed during the period April 2019 to September 2020, at two time points with a divergence of at least six months between the first (*n* = 100) and the second sampling (*n* = 98; two farms did not continue in the study). Since the formulations, feed components, and batches of the different feedstuffs varied between the two visits, both visits within each farm were treated independently (*n* = 198). The frozen basal feed samples were thawed at room temperature for 24 h and air-dried at 65 °C for 48 h. The average dry-matter content of basal feed samples was 37.06% ± 4.72% (mean ± SD, range: 25.73–54.72%). The dried samples were sequentially milled to a final particle size of ≤0.5 mm. Firstly, they were milled in the cutting mill (SM 300, Retsch GmbH, Haan, Germany) at 1500 rpm for approximately 1 min. The non-milled residues (mostly hard fragments of seeds) were subsequently milled using an ultra-centrifugal mill (ZM 200, Retsch GmbH, Haan, Germany) at 10,000 rpm for approximately 30 s. All milled fractions of each kind of sample were combined, homogeneously mixed, and packed in plastic bags (Figure 8D). Twenty grams (±0.01 g) of the whole diet representative samples was obtained by mixing proportionally milled basal and the additional concentrated feeds (supplemented based on the daily milk production) according to the average intake of each farm provided by the farmers (see Section 5.2). Then, five grams (±0.01 g) of each homogenized representative sample of the diets intended for multi-analysis was weighed in 50-mL polypropylene conical tubes (Sarstedt, Nümbrecht, Germany), and 100 g of basal feed was utilized for the chemical (proximate) analysis and stored at −20 °C until analysis.

### 5.2. Data Collection

Information regarding the kind of farming system (organic or conventional), the composition of the basal feed (major ingredients and their proportions), and total intakes of basal feeds (forage, partial, or total mixed rations), as well as the amount of additional concentrate and feed supplemented (based on the daily milk production) were obtained from those responsible for feeding management via personal interview guided by questionnaire. Per farm, the hygienic status of conserved forages and concentrates included in the rations of the lactating cows were evaluated. For the hygienic status assessment, representative samples (of at least 10 subsamples) were composited and immediately assessed. The sensory evaluation was performed considering characteristics of the appearance (colour was considered along with the presence of impurities), odour, and texture based on the methodological approaches described by Kamphues et al., 2014 [57]. The geo-climatic data, including altitude, average air temperature of the month of sampling, the average air temperature, relative humidity, and the accumulated rainfall during the growing season of maize (June–September of the previous year); average relative humidity of summer (June–September, maize’s growing season); rainfall during the summer (June–September, maize’s growing season); and averages of the air temperature of the municipalities/districts of the farms, were retrieved from the website of the Central Institution for Meteorology and Geodynamics (in German: Zentralanstalt für Meteorologie und Geodynamik—ZAMG) (available at https://www.zamg.ac.at/cms/de/klima/klimauebersichten/jahrbuch) (accessed on 1 June 2021). Summarized data are illustrated in Table 1.

### 5.3. Chemical Proximate Analysis and Particle Size Distribution of the Rations

The chemical proximate (nutrient) analysis of the samples of basal feed rations was conducted according to the protocols of the Association of German Agricultural Analytic and Research Institutes (VDLUFA, Darmstadt, Germany, 2012) [153]. The dry-matter content was determined by oven-drying the samples at 103 °C for at least 4 h (method 3.1). Ash was analysed by combustion in a muffle furnace at 550 °C overnight (method 8.1). Crude protein was determined using the Kjeldahl method (method 4.1.1) and ether extract using the Soxhlet extraction system (method 5.1.2). Analyses of NDF and the estimation of NFC were performed following the methods described by Van Soest et al. (1991) [154]. Particle size distribution of the basal rations was determined using a manually operated Penn State Particle Separator (PSPS) (model C24682N, Nasco, Fort Atkinson, WI, USA) with three sieves with aperture diameters of 19 mm, 8 mm, and 1.18 mm in diameter, according to Lammers et al. (1996) [155] and Kononoff et al. (2003) [156]. For each visit, the test was performed in duplicate, and the sieve fraction values (%) were averaged.

### 5.4. Sample Extraction and Multi-Metabolite Analysis (LC-ESI-MS/MS)

For simultaneous multi-metabolite quantification, five grams (±0.01 g) of each homogenized sample was extracted in 20 mL of the extraction solvent (acetonitrile/water/acetic acid 79:20:1, *v*/*v*/*v*) and following the procedures reported by Sulyok et al. [157]. Glacial acetic acid (p.a.) and methanol (LC gradient grade) were acquired from Merck, Darmstadt, Germany), and the water was reverse-osmosis-purified using an Elga Purelab ultra-analytic system (Veolia Water, High Wycombe, UK). Then, for sedimentation, the samples were put in a vertical position for 10–15 min. A supernatant of 500 μL of the raw extract was diluted 1:1 with a dilution solvent (acetonitrile/water/acetic acid 20:79:1, *v*/*v*/*v*) in vials. The injection volume of both raw extracts of the samples and standard solutions of the analytes was 5 μL. These volumes were put into the QTrap 5500 LC-MS/MS system (Applied Biosystems, Foster City, CA, USA) equipped with a TurboV electrospray ionization (ESI) source, which was coupled to a 1290 series UHPLC system (Agilent Technologies, Waldbronn, Germany) as described by Sulyok et al., 2020 [157]. A subsequent quantification from external calibration by serial dilutions of a stock solution of analysed compounds was completed. Finally, the results were adjusted for apparent recoveries defined through spiking experiments according to Steiner [158]. Standards of fungal, plant, and unspecific secondary metabolites were purchased from several commercial suppliers or obtained via a donation from different research institutions [157,158]. This analytical methodology has been validated [157,158] and has been employed to study multi-mycotoxin occurrence in complex feedstuff matrices such as silage, pastures, concentrate feed, and total mix rations [53,61,159,160]. The accuracy of the method is verified on a routine basis by participation in proficiency testing organized by BIPEA (Genneviliers, France). Satisfactory z-scores between −2 and 2 have been obtained for >95% of the >1700 results submitted so far. In particular, 17 out of 18 results submitted for a sample of MS were in this range, the exception being zearalenone exhibiting z = −2.05.

### 5.5. Statistical Analysis

Frequencies of contamination (occurrences) and the descriptive statistics of the concentrations of metabolites (average, SD, median, and range values) were calculated considering values over the limit of detection (LOD). Values lower than the limit of quantification (LOQ) were processed as LOQ/2. Concentrations of metabolites are expressed in μg/kg parts per billion (ppb) on a dry-matter basis and plotted on a logarithmic scale (Log_10_) where applicable. The co-occurrence analyses of mycotoxins and plant metabolites were performed separately using Microsoft Excel, constructing matrices that included metabolites with detection frequencies over 20%. Spearman’s correlation coefficients were computed, and heatmaps were plotted using GraphPad Prism (Prism version 9.1, GraphPad Software, San Diego, CA, USA). The correlation analysis was interpreted considering only significative correlations with ρ ≥ 0.3, based on Hinkle et al. (2003) [161]. Multiple regression analysis was performed using SAS (version 9.4; SAS Institute Inc., Cary, NC, USA) to investigate the influences of dietary and geoclimatic factors on dependent variables of interest: concentrations of total metabolites produced by fungi, plants, *Alternaria*, *Aspergillus*, *Fusarium*, *Penicillium*, EAs, DON, ZEN, FUM (the sum of FB1 and FB2), BEA, ENNs, CUL, siccanol, and phytoestrogens. Data were log-transformed to normalize the data. For some variables (DON, FUM, siccanol, total *Alternaria* metabolites, total *Penicillium* metabolites, and total fungal metabolites), extreme data that still led to screwed data were manually excluded. The normality of data based on the Shapiro–Wilk test (*p* > 0.05) and Q-Q-plot were ensured before further data analysis. For each dependent variable, a set of independent variables including dietary proportions of MS, straw, hay, BSG, feed particle size > 19 mm and between 8–1.18 mm, the content of crude protein, ash, ether extract, ash, and non-fibre carbohydrate, the hygienic status of MS and GS, altitude, temperature, relative humidity, and rainfall were tested, and the candidate independent variables were selected based on a step-wise selection using the procedure SELECT of SAS. All candidate independent variables passed the collinearity test, having a variance inflation factor less than 10. Next, the effects of candidate variables, including their squared terms and interactions, were investigated using the mixed procedure of SAS. The model also included the random effect of two rounds of visits. Backward elimination was performed to obtain the final model using the protocol described previously [162]. Additionally, *R^2^* and RMSE of the final model were calculated. In addition, the odds ratio and predicted probabilities for high contamination of *Fusarium* metabolites due to the inclusion levels of forage sources were determined using PROC LOGISTIC (SAS version 9.4; SAS Institute Inc., Cary, NC, USA). For this analysis, data classified as low (25 percentile, *n* = 49) and high *Fusarium* metabolite concentrations (75 percentile, *n* = 60) were used. The model included dietary levels of MS, GS, straw, hay, BGS, other silages, and quadratic terms of MS, because they were found to show significance in multiple regression analysis.

## Figures and Tables

**Table 1:** Potential factors influencing the levels of fungal (toxic) metabolites and phytoestrogens: Characteristics of the rations of lactating Austrian dairy cows, the hygienic status of the main ingredients, and geo-climatic parameters of farms’ locations.

Dietary Related Factors				
**Dietary Component**	**Farm Frequency of Inclusion (%)**		**Average ± SD**	**Range**
Grass silage (%DM)	97.5		40.4 ± 16.3	10.4–86.7
Maize silage (%DM)	82.8		22.4 ± 14.3	1.7–59.1
Hay (%DM)	18.2		0.9 ± 3.2	0.6–29.8
Straw (%DM)	62.1		1.8 ± 2.1	0.01–10.0
BSG (%DM)	27.3		4.11 ± 2.4	0.34–13.5
Other silages (%DM)	10.1		6.29 ± 5.67	0.47–23.6
Forage (%DM)	100		65.9 ± 10.1	32.4–89
**Chemical composition**				
Dry matter (%)			37.1 ± 4.7	25.7–54.6
Crude protein (%DM)			15.4 ± 2.0	9.9–21.2
Ash (%DM)			8.2 ± 2.5	4.8–18.5
Crude fat (%DM)			2.7 ± 0.5	1.2–4.6
Neutral detergent fibre (% DM)			50.4 ± 7.0	36.8–75.2
Non-fibre carbohydrate (% DM)			23.3 ± 7.3	0.8–41.3
**Particle size**				
>19 mm (%)			46.8 ± 19.8	2.3–96.0
8–19 mm (%)			22.7 ± 11.2	2–53.6
1.18–8 mm (%)			25.6 ± 9.3	1.6–49.0
<1.18 mm (%)			4.6 ± 2.9	0.3–13.7
**Hygienic status**	**Proper**	**Minor** **deficiency**	**Significant deficiency**	**Vast deficiency**
Grass silage (%)	54.9	27.5	9.8	7.8
Maize silage (%)	45.7	43.9	3.7	6.7
Hay (%)	91.7	5.6	2.8	0
Straw (%)	80.5	17.1	1.6	0.8
BSG (%)	55.6	37	1.9	5.6
Concentrate (%)	97	1	1	1
**Geo-climatic factors**				
			**Average ± SD**	**Range**
Altitude (m.a.s.l.)			480.3 ± 162.1	262–1300
Temperature (mean month of sampling) (°C) ^a^			15.47 ± 6.19	−0.8–22.4
Temperature (maize’s growing season) (°C) ^b^			18.7 ± 1.1	13–22
Relative humidity (%) ^c^			70.1 ± 3.3	60.3–78
Rainfall (mm) ^d^			294.5 ± 60.3	179–594

^a^ average temperature of the month of sampling; ^b^ average temperature of summer (June–September, maize’s growing season); ^c^ average relative humidity of summer (June–September, maize’s growing season); ^d^ rainfall during the summer (June–September, maize’s growing season).

**Table 2:** Occurrence and concentration of mycotoxins, phytoestrogens, and other fungal, plant, and unspecific secondary metabolites detected in representative samples of whole diets of lactating cows (*n* = 198) from Austria.

Group	Metabolite	Positive Samples (%) ^1^	Concentration (µg/kg DM) ^2^						
			Average ± SD			Median	Range		
*Alternaria*	Alternariol ^3^	45.5	8.55	±	13.8	5.65	1.09	–	118
	Alternariolmethylether ^3^	42.4	5.69	±	3.6	5.50	1.07	–	20.0
	Altenuisol	1.0	15.3	±	5.3	15.3	9.96	–	20.6
	Altersetin	47.0	34.3	±	26.4	26.4	4.16	–	143
	Infectopyrone	78.3	348	±	490	169	6.96	–	3810
	Pyrenophorol	2.5	8.31	±	9.6	4.05	1.90	–	27.5
	Radicinin	1.0	4.44	±	2.7	4.44	1.72	–	7.17
	Tentoxin	30.8	3.79	±	2.2	3.41	1.15	–	12.1
	Tenuazonic acid ^3^	78.8	178	±	83.1	153	76.1	–	549
	Zinndiol	1.0	19.8	±	1.9	19.8	17.9	–	21.7
	Zinniol	2.5	42.0	±	23.7	36.4	22.4	–	87.6
	Total ^4^	98.5	445	±	491	304	2.62	–	3930
*Aspergillus*	Aflatoxin B1 ^5^	0		-		-		-	
	Averufin	7.6	2.69	±	1.6	2.95	1.07	–	8.03
	Bis(methylthio)gliotoxin	4.0	12.8	±	6.5	11.9	5.67	–	25.7
	Deoxygerfelin	2.0	9.37	±	11.5	3.84	0.75	–	29.0
	Deoxynortryptoquivalin	1.5	3.20	±	0.0	3.20	3.20	–	3.20
	Flavoglaucin	75.8	21.4	±	54.2	5.94	0.65	–	368
	Fumigaclavine	1.0	6.08	±	1.1	6.08	5.00	–	7.15
	Fumigaclavine C	2.0	28.4	±	20.9	24.2	6.52	–	58.6
	Fumiquinazolin D	2.0	26.7	±	11.5	25.8	14.3	–	40.9
	Integracin A	7.1	23.1	±	69.8	1.95	1.11	–	275
	Integracin B	11.6	50.7	±	219	2.87	1.05	–	1080
	Kojic acid	56.1	165	±	62.2	145	132	–	516
	Methylsulochrin	1.0	18.2	±	1.9	18.2	16.3	–	20.1
	Mevinolin	14.1	36.1	±	35.2	23.8	12.0	–	150
	Sterigmatocystin ^3^	17.2	3.60	±	2.3	2.65	1.19	–	10.3
	Trypacidin	0.5		-		-	2.78		
	Versicolorin C	2.5	5.80	±	3.3	7.60	1.75	–	9.7
	Total ^4^	88.4	141	±	159	150	1.03	–	1680
Ergot alkaloids	Chanoclavine	18.2	7.90	±	12.0	3.23	0.95	–	55.8
	Festuclavine	1.0	11.4	±	8.6	11.4	2.75	–	20.0
	Ergocornine	9.1	6.43	±	7.7	4.32	1.26	–	34.8
	Ergocorninine	5.6	5.53	±	5.7	3.38	1.60	–	22.1
	Ergocristine	4.5	7.86	±	3.4	6.90	1.90	–	13.5
	Ergocristinine	2.5	5.12	±	2.8	4.14	1.35	–	8.53
	Ergocryptine	8.6	10.6	±	11.0	7.21	0.95	–	43.2
	Ergocryptinine	2.0	8.94	±	4.0	9.63	3.49	–	13.0
	Ergometrine	0.5		-		-	7.18		
	Ergosine	9.1	6.76	±	4.8	5.43	0.30	–	17.2
	Ergosinine	8.6	5.01	±	6.1	2.90	0.30	–	24.5
	Ergotamine	6.6	9.64	±	15.4	4.94	1.61	–	62.3
	Ergotaminine	6.1	9.19	±	14.4	3.99	2.00	–	56.2
	Total ^4^	32.3	19.5	±	37.3	8.01	0.95	–	219
*Fusarium*	15-Hydroxyculmorin ^3,6^	94.4	128	±	156	87.3	10.6	–	1600
	Acuminatum B	7.6	47.5	±	18.7	38.8	23.2	–	80.7
	Antibiotic Y	40.4	35.1	±	33.1	24.4	8.52	–	175
	Apicidin ^3^	75.8	16.1	±	15.0	12.2	0.75	–	105
	Apicidin D2	8.1	14.7	±	13.1	6.95	6.95	–	57.2
	Aurofusarin ^3^	96.0	59.3	±	42.3	46.9	6.79	–	349
	Beauvericin ^3^	100	10.3	±	9.1	7.38	0.98	–	71.7
	Bikaverin ^3^	66.2	25.6	±	24.6	18.4	3.83	–	161
	Chrysogine	8.6	32.0	±	33.6	23.9	1.68	–	136
	Culmorin ^3^	92.4	361	±	324	272	35.3	–	2952
	Deoxynivalenol (5000) ^5^	92.4	153	±	230	104	14.8	–	2900
	DON-3-glucoside ^6^	9.1	33.9	±	41.0	19.0	19.0	–	195
	Enniatin A ^3^	65.2	1.79	±	3.2	1.07	0.20	–	31.1
	Enniatin A1 ^3^	99.5	6.92	±	5.7	5.28	0.40	–	32.3
	Enniatin B ^3^	100	40.2	±	28.1	31.4	4.34	–	175
	Enniatin B1 ^3^	100	25.9	±	18.7	21.2	2.42	–	126
	Enniatin B2 ^3^	69.2	1.34	±	0.9	1.07	0.22	–	6.81
	Epiequisetin ^3^	53.5	5.08	±	8.2	3.07	1.07	–	63.4
	Equisetin^3^	97.0	13.4	±	22.3	7.73	1.60	–	224
	Fumonisin A1 (precussor)	1.0	3.97	±	0.4	3.97	3.62	–	4.32
	Fumonisin B1 ^5^	70.7	120	±	118	93.5	26.5	–	1120
	Fumonisin B2 ^5^	35.4	51.9	±	32.9	45.3	17.0	–	243
	Fumonisin B3	6.1	43.3	±	29.4	26.5	19.9	–	129
	Fumonisin B4	4.5	33.9	±	24.9	18.0	18.0	–	96.9
	Fusaproliferin	4.5	184	±	76.8	174	81.6	–	338
	Fusapyron ^3^	2.0	10.9	±	9.6	6.42	3.49	–	27.5
	HT-2 glucoside ^6^	1.0	14.4	±	8.4	14.4	6.00	–	22.7
	HT-2 toxin ^5^	27.8	27.3	±	28.2	20.5	9.27	–	217
	Moniliformin ^3^	40.4	23.4	±	22.4	16.1	4.61	–	148
	Monoacetoxyscirpenol	6.6	13.6	±	7.6	11.0	5.52	–	29.5
	Nivalenol	8.6	311	±	247	269	34.6	–	804
	Siccanol ^3^	54.0	709	±	805	494	106	–	7220
	T-2 toxin ^5^	12.1	4.97	±	2.5	4.25	2.13	–	14.6
	W493	65.7	21.7	±	69.9	5.64	1.00	–	671
	Zearalenone (500) ^5^	77.8	25.2	±	36.9	14.7	1.90	–	378
	Sum of enniatins	100	75.0	±	50.4	61.1	7.36	–	324
	Sum of T-2 and HT-2 toxins (250) ^5^	32.3	25.3	±	27.4	20.4	2.13	–	217
	Sum of fumonisins	71.2	150	±	169	106	26.5	–	1590
	Sum of fumonisins B1 and B2 (50,000) ^5^	71.2	145	±	149	102	26.5	–	1370
	Sum of type A trichothecenes	36.9	25.0	±	29.8	19.0	2.13	–	246
	Sum of type B trichothecenes	92.9	184	±	266	113	14.8	–	3070
	Total ^4^	100	1390	±	1510	1070	109	–	17,800
*Penicillium*	7-Hydroxypestalotin	3.0	4.39	±	2.6	2.60	2.60	–	9.07
	Andrastin A	16.7	25.8	±	33.7	12.0	1.80	–	140
	Andrastin B	4.0	68.8	±	66.6	48.4	16.5	–	238
	Andrastin C	3.5	270	±	170	247	43.4	–	603
	Barceloneic acid	18.2	36.4	±	29.9	24.7	7.84	–	133
	Citreohybridinol	1.0	3.77	±	1.6	3.77	2.16	–	5.38
	Citrinin	1.0	20.7	±	14.0	20.7	6.67	–	34.7
	Curvularin	6.1	49.7	±	64.3	14.9	2.54	–	182
	Dehydrocurvularin	1.5	54.5	±	35.2	32.8	26.5	–	104
	Fellutanine A	93.9	96.3	±	62.5	78.9	27.7	–	466
	Griseofulvin	0.5		-		-	1.83		
	Hydroxyandrastin C	3.0	10.8	±	7.0	9.41	3.10	–	20.8
	Marcfortine A	23.2	9.49	±	15.4	3.88	0.45	–	81.0
	Marcfortine C	6.1	3.08	±	3.2	1.57	0.45	–	12.1
	Mycophenolic acid ^3^	21.2	47.5	±	104	15.8	1.52	–	661
	Ochratoxin A (250) ^5^	1.0	7.50	±	0.3	7.50	7.16	–	7.84
	Pestalotin	14.1	5.59	±	2.8	3.30	1.88	–	11.3
	Phenopyrrozin	96.5	52.8	±	36.8	42.7	10.8	–	352
	Questiomycin A	5.1	27.1	±	14.1	20.8	11.1	–	59.5
	Questiomycin Derivat	18.7	58.4	±	153	32.6	9.82	–	973
	Questiomycine	36.4	8.17	±	9.3	5.23	1.50	–	49.2
	Roquefortine C	18.7	30.3	±	64.7	14.5	3.56	–	387
	Roquefortine D	1.5	9.69	±	7.7	4.25	4.25	–	20.6
	Total ^4^	99.5	205	±	176	166	2.71	–	1680
Lichen- associated fungi	Lecanoric acid	6.1	4.71	±	6.0	1.45	1.45	–	18.1
	Usnic acid	11.6	3.83	±	3.2	2.53	0.50	–	12.7
	Total ^4^	16.2	4.57	±	4.9	2.47	0.50	–	18.9
Other fungi	Alamethicine	1.5	65.5	±	40.1	61.2	18.8	–	117
	Ascochlorin	9.6	3.35	±	3.3	2.07	1.15	–	13.6
	Ascofuranone	0.5		-		-	3.57		
	Bassianolide	2.0	8.25	±	9.4	3.90	0.80	–	24.4
	Calphostin C	3.0	2.89	±	2.5	1.98	1.09	–	8.34
	Cytochalasin B	13.1	48.3	±	51.2	34.6	8.87	–	234
	Cytochalasin C	1.0	8.77	±	0.9	8.77	7.90	–	9.6
	Destruxin B	27.3	5.66	±	7.5	3.26	0.20	–	44.1
	Emestrin	3.5	16.2	±	11.3	22.3	3.50	–	31.0
	Epoxycytochalsin C	7.6	3.59	±	3.7	0.60	0.60	–	12.2
	Ilicicolin A	13.1	2.53	±	2.8	1.42	0.50	–	10.1
	Ilicicolin B	38.9	4.79	±	6.5	1.89	1.02	–	36.5
	Ilicicolin E	5.6	4.53	±	3.0	3.93	0.50	–	10.2
	Ilicicolin H	22.2	16.1	±	20.8	10.5	0.50	–	123
	LL-Z 1272e	1.5	10.4	±	8.3	8.89	1.03	–	21.3
	Monocerin	33.3	68.1	±	162	11.9	0.65	–	893
	Myriocin	1.0	41.4	±	24.0	41.4	17.4	–	65.3
	Rubellin D	57.1	34.8	±	54.2	15.5	0.85	–	301
	Neoechinulin A	35.9	27.6	±	54.2	17.7	2.00	–	429
	Sporidesmolide II	51.5	65.8	±	114	23.6	0.25	–	617
	Ternatin	1.5	7.59	±	6.5	6.39	0.25	–	16.1
	Total ^4^	89.9	115	±	177	45.1	1.15	–	1060
Sum of fungal metabolites		100	2260	±	1690	1993	302	–	19,100
Phytoestrogens	Biochanin	100	21,900	±	15,800	23,000	226	–	52,050
	Coumestrol	80.8	524	±	1140	111	2.50	–	8290
	Daidzein	99.5	5780	±	6670	3110	25.0	–	45,900
	Daidzin	89.9	4527	±	4580	3300	3.38	–	23,900
	Formonetin	21.2	78,700	±	67,900	58,400	13,800	–	289,000
	Genistein	100	9460	±	8950	6730	179	–	52,600
	Genistin	93.4	6000	±	6130	3980	33.0	–	36,500
	Glycitein	53.0	9430	±	10,200	4530	138	–	48,100
	Glycitin	80.8	1205	±	1160	930	12.5	–	7540
	Ononin	73.7	435	±	1050	160	14.0	–	11,540
	Total ^4^	100	70,200	±	67,100	50,800	1080	–	411,000
Other plant metabolites	Abscisic acid	89.4	785	±	552	627	136	–	4315
	Chaconin	11.6	31.4	±	41.3	7.50	5.60	–	161
	Colchicine	3.5	71.2	±	87.9	31.6	13.5	–	282
	Linamarin	47.0	2850	±	2860	1520	82.5	–	14,200
	Lotaustralin	74.2	1300	±	2160	558	18.1	–	13,700
	Xanthotoxin	62.6	37.4	±	74.2	10.9	0.90	–	450
	Total ^4^	98.0	3090	±	4260	1522	5.37	–	24,400
Sum of plant metabolites		100	73,500	±	67,300	54,500	1204	–	413,000
Unspecific	3-Nitropropionic acid	8.1	43.4	±	41.2	20.8	10.7	–	158
	Asperglaucide	72.7	5.82	±	12.5	3.23	0.60	–	136
	Asperphenamate	69.2	8.41	±	24.1	2.64	0.50	–	216
	Brevianamid F	100	264	±	147	256	17.0	–	899
	Chrysophanol	53.5	576	±	1390	276	19.0	–	12,500
	Citreorosein	18.2	178	±	197	108	28.3	–	954
	cyclo(L-Pro-L-Tyr)	100	4100	±	2320	3720	569	–	15,400
	cyclo(L-Pro-L-Val)	100	13,700	±	6390	12,780	2720	–	36,900
	Emodin	97.0	249	±	355	92.4	4.26	–	1957
	Endocrocin	9.6	292	±	255	215	40.5	–	1090
	Iso-Rhodoptilometrin	45.5	2.07	±	2.1	1.22	0.40	–	9.01
	N-Benzoyl-Phenylalanine	5.6	28.9	±	37.8	14.2	1.00	–	111
	Norlichexanthone	25.8	20.2	±	103	0.55	0.55	–	745
	Oxyskyrin	0.5		-		-	6.36		
	Physcion	20.2	844	±	683	655	49.7	–	2560
	Rugulusovine	100	271	±	132	257	19.6	–	817
	Skyrin	49.0	4.2	±	4.1	2.92	0.15	–	28.8
	Tryptophol	77.8	1030	±	1200	564	49.2	–	6380
Sum of unspecific metabolites		100	20,000	±	8870	18,600	3740	–	52,400
Sum of all detected metabolites		100	95,400	±	68,900	78,300	15,100	–	432,000

^1^ with values > limit of detection (LOD); ^2^ computations performed without data < LOD. In case values > LOD and < limit of quantification (LOQ), LOQ/2 was used for calculation; ^3^ classified as emerging mycotoxins [60,61,62], ^4^ accumulative values of occurrences and concentrations of all the metabolites belonging to the group, ^5^ classified as regulated mycotoxins and their respective maximum level (for AFB1) and guidance levels (for the other mycotoxins) expressed in µg/kg for a dairy cattle feedstuff with a moisture content of 12% (European Commission, 2002, 2006, 2012) [14,15,16,17], and ^6^ modified mycotoxins [63].

**Table 3:** Influences of the dietary parameters and geo-climatic factor on the concentration of mycotoxins, fungal metabolites, and phytoestrogens.

Concentration (Log-µg/kg)	*n*	Intercept	SE	*p* Value	Influencing Factors	Coefficients	SE	*p* Value	R^2^	RMSE
*Alternaria* metabolites	190	5.2607	0.0821	<0.001	Straw	+0.3851	0.0616	<0.001	0.26	0.757
					Straw × Straw	−0.0282	0.0082	<0.001		
*Fusarium* metabolites	198	6.2526	0.5082	<0.001	MS	+0.0695	0.0147	<0.001	0.52	0.579
					Sieve > 19 mm	−0.0158	0.0072	0.030		
					Straw	−0.5902	0.1714	<0.001		
					NFC	−0.0413	0.0174	0.019		
					MS × MS	−0.00082	0.0002	<0.001		
					MS × Straw	+0.00398	0.0019	0.042		
					MS × Sieve > 19 mm	−0.00041	0.0002	0.016		
					Straw × NFC	+0.02352	0.0069	<0.001		
					Straw × Sieve > 19 mm	+0.01151	0.0031	<0.001		
					NFC× Sieve > 19 mm	+0.00064	0.0003	0.047		
					Straw × NFC × Sieve > 19 mm	−0.00047	0.0001	<0.001		
Deoxynivalenol	182	5.7616	0.8443	<0.001	MS	+0.09058	0.0247	<0.001	0.22	0.677
					Rainfall	−0.01240	0.0049	0.013		
					MS × Rainfall	−0.00017	0.0001	0.031		
					MS × MS	−0.00057	0.0002	0.010		
					Rainfall × Rainfall	+0.000022	0.0000	0.009		
Zearalenone	154	0.9462	0.5023	0.057	EE	+0.3897	0.1421	0.007	0.22	0.918
					Sieve > 19 mm	−0.0124	0.0041	0.003		
					Hygiene GS	+0.2147	0.0745	0.004		
Fumonisins B1 and B2	125	4.6964	0.110	<0.001	Straw	−0.07163	0.0278	0.011	0.09	0.606
					Hygiene MS	+0.1470	0.0585	0.013		
Beauvericin	198	−1.3010	0.6717	0.054	MS	+0.0152	0.0037	<0.001	0.32	0.654
					Sieve 1.18–8 mm	+0.0198	0.0055	<0.001		
					Crop temperature	+0.1439	0.0374	<0.001		
Culmorin	183	4.4483	0.3138	<0.001	MS	+0.06254	0.0157	<0.001	0.34	0.611
					Sieve > 19 mm	−0.00234	0.0046	0.611		
					MS × MS	−0.00072	0.0002	0.001		
					MS × Sieve > 19 mm	−0.00039	0.0002	0.025		
Enniatins	198	3.5175	0.1333	<0.001	Brewery’s spent grains	+0.1111	0.0192	<0.001	0.19	0.600
Siccanol	107	7.0348	0.4132	<0.001	MS	+0.0016	0.0067	0.808	0.30	0.627
					Sieve > 19 mm	−0.0098	0.0033	0.003		
					Straw	−0.0487	0.0524	0.353		
					MS × Straw	+0.0052	0.0021	0.016		
*Penicillium* metabolites	187	3.9964	0.2731	<0.001	Temp sampling	+0.0726	0.0233	0.002	0.12	0.483
					Forage	+0.1135	0.0035	0.002		
					Temp sampling × Temp sampling	−0.0024	0.0097	0.013		
Total fungal metabolites	190	9.0404	0.7690	<0.001	MS	−0.0118	0.0012	0.345	0.44	0.408
					Straw	+0.0864	0.0509	0.091		
					Ash	−0.3912	0.1364	0.005		
					NFC	−0.0148	0.0055	0.008		
					Sieve > 19 mm	+0.0003	0.0035	0.932		
					Hygiene GS	−0.0633	0.0314	0.045		
					Ash × Ash	+0.01395	0.0056	0.014		
					Straw × Straw	−0.01337	0.0046	0.004		
					Straw × Sieve > 19 mm	+0.00185	0.0008	0.019		
					MS × Sieve > 19 mm	−0.00031	0.0001	0.011		
					MS × Ash	+0.00421	0.0017	0.015		

SE = standard error; RMSE = root mean square error; MS = proportion of maize silage in the diets; Straw = proportion of straw in the diets; Ash = proportion of ash in the mixed rations; NFC = proportion of non-fibre carbohydrates in the mixed rations; EE = proportion of etheric extract in the mixed rations; Sieve > 19 mm = proportion of feed particles with diameter longer than 19 mm in the diets; Temp sampling = temperature at the sampling month; Crop temperature = average temperature of summer (June–September, maize’s growing season); Rainfall = accumulated rainfall (mm) during the summer (June–September, maize’s growing season); Humidity crop = average relative humidity (%) of summer (June–September, maize’s growing season); Hygiene MS = hygienic score of maize silage; Hygiene GS = hygienic score of grass silage.

## Data Availability

Data available on request due to restrictions (Data protection agreement).

## Supplementary Materials

The following are available online at https://www.mdpi.com/article/10.3390/toxins14070493/s1. Table S1. List of 863 targeted metabolites via a validated multi-metabolite liquid chromatography/electrospray ionization–tandem mass spectrometric (LC/ESI–MS/MS) method. Table S2. Odds ratio estimates and profile-likelihood confidence intervals of forage inclusion levels as dietary risk factors for high *Fusarium* mycotoxin loads (above 75th percentile concentrations). Figure S1. Predicted probabilities for *Fusarium* mycotoxin loads (above 75th percentile concentrations) related to the proportion of (a) maize silage and (b) straw in the dietary rations of Austrian dairy cows.

## References

[1] BMLRT (Bundesministerium für Landwirtschaft, Regionen und Tourismus) Grüner Bericht 2021. Die Situation der österreichischen Land- und Forstwirtschaft. BMLRT, Vienna. https://gruenerbericht.at/cm4/jdownload/send/2-gr-bericht-terreich/2393-gb2021.

[2] FAO, IDF, IFCN (2014). World Mapping of Animal Feeding Systems in the Dairy Sector.

[3] Webster J. (2020). Understanding the Dairy Cow.

[4] Mayne C., Gordon F. (1984). The effect of type of concentrate and level of concentrate feeding on milk production. Anim. Sci..

[5] Sairanen A., Khalili H., Virkajärvi P. (2006). Concentrate supplementation responses of the pasture-fed dairy cow. Livest. Sci..

[6] Gallo A., Giuberti G., Frisvad J.C., Bertuzzi T., Nielsen K.F. (2015). Review on Mycotoxin Issues in Ruminants: Occurrence in Forages, Effects of Mycotoxin Ingestion on Health Status and Animal Performance and Practical Strategies to Counteract Their Negative Effects. Toxins.

[7] Santos Pereira C., Cunha S.C., Fernandes J.O. (2019). Prevalent mycotoxins in animal feed: Occurrence and analytical methods. Toxins.

[8] Fletcher M.T., Netzel G. (2020). Food Safety and Natural Toxins. Toxins.

[9] Reed K.F.M. (2016). Fertility of herbivores consuming phytoestrogen-containing *Medicago* and *Trifolium* species. Agriculture.

[10] FAO, WHO (2019). Hazards Associated with Animal Feed. https://www.fao.org/3/ca6825en/CA6825EN.pdf.

[11] Bryden W.L. (2012). Mycotoxin contamination of the feed supply chain: Implications for animal productivity and feed security. Anim. Feed Sci. Technol..

[12] Fink-Gremmels J. (2005). Mycotoxins in forages. The Mycotoxin Blue Book.

[13] CAST (2003). Mycotoxins: Risks in Plant, Animal and Human Systems, Report No. 139.

[14] European Commission (2006). Recommendation of 17 August 2006 on the presence of deoxynivalenol, zearalenone, ochratoxin A, T-2 and HT-2 and fumonisins in products intended for animal feeding (2006/576/EC). Off. J. Eur. Union..

[15] European Commission (2002). Directive 2002/32/EC of the European Parliament and of the Council of 7 May 2002 on undesirable substances in animal feed. Luxemb. Off. J. Eur. Union..

[16] European Commission (2013). Commission Recommendation of 27 March 2013 on the presence of T-2 and HT-2 toxin in cereals and cereal products (2013/165/EU). Off. J. Eur. Union..

[17] European Commission (2012). Commission recommendation 2012/154/EU of 15 March 2012 on the monitoring of the presence of ergot alkaloids in feed and food. Off. J. Eur. Union..

[18] EFSA (2013). Scientific Opinion on the risk for public and animal health related to the presence of sterigmatocystin in food and feed. EFSA J..

[19] EFSA (2014). Scientific Opinion on the risks to human and animal health related to the presence of beauvericin and enniatins in food and feed. EFSA J..

[20] Jestoi M. (2008). Emerging *Fusarium*-mycotoxins fusaproliferin, beauvericin, enniatins, and moniliformin—A review. Crit. Rev. Food. Sci. Nutr..

[21] Křížová L., Dadáková K., Dvořáčková M., Kašparovský T. (2021). Feedborne Mycotoxins Beauvericin and Enniatins and Livestock Animals. Toxins.

[22] Panasiuk L., Jedziniak P., Pietruszka K., Piatkowska M., Bocian L. (2019). Frequency and levels of regulated and emerging mycotoxins in silage in Poland. Mycotoxin Res..

[23] Zachariasova M., Dzuman Z., Veprikova Z., Hajkova K., Jiru M., Vaclavikova M., Zachariasova A., Pospichalova M., Florian M., Hajslova J. (2014). Occurrence of multiple mycotoxins in european feedingstuffs, assessment of dietary intake by farm animals. Anim. Feed Sci. Technol..

[24] Battilani P., Palumbo R., Giorni P., Dall’Asta C., Dellafiora L., Gkrillas A., Toscano P., Crisci A., Brera C., De Santis B. (2020). Mycotoxin mixtures in food and feed: Holistic, innovative, flexible risk assessment modelling approach: MYCHIF. EFSA Support. Publ..

[25] Smith M.-C., Madec S., Coton E., Hymery N. (2016). Natural co-occurrence of mycotoxins in foods and feeds and their in vitro combined toxicological effects. Toxins.

[26] Speijers G.J.A., Speijers M.H.M. (2004). Combined toxic effects of mycotoxins. Toxicol. Lett..

[27] McAllister T.A., Ribeiro G., Stanford K., Wang Y. (2020). Forage-Induced Animal Disorders. Forages.

[28] Wolawek-Potocka I., Bah M.M., Korzekwa A., Piskula M.K., Wiczkowski W., Depta A., Skarzynski D.J. (2005). Soybean-derived phytoestrogens regulate prostaglandin secretion in endometrium during cattle estrous cycle and early pregnancy. Exp. Biol. Med..

[29] Wocławek-Potocka I., Korzekwa A., Skarzyński D.J. (2008). Can phytoestrogens pose a danger in the reproduction of cows?. Med. Weter..

[30] Wocławek-Potocka I., Mannelli C., Boruszewska D., Kowalczyk-Zieba I., Waśniewski T., Skarżyński D.J. (2013). Diverse effects of phytoestrogens on the reproductive performance: Cow as a model. Int. J. Endocrinol..

[31] Romero-R C.M., Castellanos M.d.R.T., Mendoza R.M., Reyes R.A., García A.R. (1997). Oestrogenic syndrome in dairy cows by alfalfa comsuption with large amount of coumestrol. Vet. Mex..

[32] Vejdovszky K., Schmidt V., Warth B., Marko D. (2017). Combinatory estrogenic effects between the isoflavone genistein and the mycotoxins zearalenone and alternariol in vitro. Mol. Nutr. Food Res..

[33] Vejdovszky K., Hahn K., Braun D., Warth B., Marko D. (2017). Synergistic estrogenic effects of Fusarium and Alternaria mycotoxins in vitro. Arch. Toxicol..

[34] Hessenberger S., Botzi K., Degrassi C., Kovalsky P., Schwab C., Schatzmayr D., Schatzmayr G., Fink-Gremmels J. (2017). Interactions between plant-derived oestrogenic substances and the mycoestrogen zearalenone in a bioassay with MCF-7 cells. Pol. J. Vet. Sci..

[35] Martins C., Vidal A., De Boevre M., Assunção R., Zaragoza O., Casadevall A. (2021). Mycotoxins as Endocrine Disruptors–An Emerging Threat. Encyclopedia of Mycology.

[36] Johny A., Fæste C.K., Bogevik A.S., Berge G.M., Fernandes J.M., Ivanova L. (2019). Development and validation of a liquid chromatography high-resolution mass spectrometry method for the simultaneous determination of mycotoxins and phytoestrogens in plant-based fish feed and exposed fish. Toxins.

[37] Socas-Rodríguez B., Lanková D., Urbancová K., Krtková V., Hernández-Borges J., Rodríguez-Delgado M.Á., Pulkrabová J., Hajšlová J. (2017). Multiclass analytical method for the determination of natural/synthetic steroid hormones, phytoestrogens, and mycoestrogens in milk and yogurt. Anal. Bioanal. Chem..

[38] Grgic D., Varga E., Novak B., Müller A., Marko D. (2021). Isoflavones in Animals: Metabolism and Effects in Livestock and Occurrence in Feed. Toxins.

[39] Daou R., Joubrane K., Maroun R.G., Khabbaz L.R., Ismail A., El Khoury A. (2021). Mycotoxins: Factors influencing production and control strategies. AIMS Agric. Food..

[40] Yang L., Wen K.-S., Ruan X., Zhao Y.-X., Wei F., Wang Q. (2018). Response of plant secondary metabolites to environmental factors. Molecules.

[41] Pavarini D.P., Pavarini S.P., Niehues M., Lopes N.P. (2012). Exogenous influences on plant secondary metabolite levels. Anim. Feed Sci. Technol..

[42] Penagos-Tabares F., Khiaosa-ard R., Nagl V., Faas J., Jenkins T., Sulyok M., Zebeli Q. (2021). Mycotoxins, Phytoestrogens, and Other Secondary Metabolites in Austrian Pastures: Occurrences, Contamination Levels, and Implications of Geo-climatic Factors. Toxins.

[43] Ramirez M.L., Chulze S., Magan N. (2004). Impact of environmental factors and fungicides on growth and deoxinivalenol production by *Fusarium graminearum* isolates from Argentinian wheat. Crop Prot..

[44] Bernhoft A., Torp M., Clasen P.-E., Løes A.-K., Kristoffersen A. (2012). Influence of agronomic and climatic factors on *Fusarium* infestation and mycotoxin contamination of cereals in Norway. Food Addit. Contam. Part A Chem. Anal. Control Expo. Risk Assess..

[45] Bernhoft A., Clasen P.-E., Kristoffersen A., Torp M. (2010). Less *Fusarium* infestation and mycotoxin contamination in organic than in conventional cereals. Food Addit. Contam. Part A Chem. Anal. Control Expo. Risk Assess..

[46] Perrone G., Ferrara M., Medina A., Pascale M., Magan N. (2020). Toxigenic fungi and mycotoxins in a climate change scenario: Ecology, genomics, distribution, prediction and prevention of the risk. Microorganisms.

[47] Nichea M.J., Cendoya E., Zachetti V.G.L., Chiacchiera S.M., Sulyok M., Krska R., Torres A.M., Chulze S.N., Ramirez M.L. (2015). Mycotoxin profile of *Fusarium armeniacum* isolated from natural grasses intended for cattle feed. World Mycotoxin J..

[48] Rasmussen R.R., Storm I., Rasmussen P.H., Smedsgaard J., Nielsen K.F. (2010). Multi-mycotoxin analysis of maize silage by LC-MS/MS. Anal. Bioana. Chem..

[49] Storm I., Rasmussen R.R., Rasmussen P.H. (2014). Occurrence of Pre- and Post-Harvest Mycotoxins and Other Secondary Metabolites in Danish Maize Silage. Toxins.

[50] Rodriguez-Blanco M., Marin S., Sanchis V., Ramos A.J. (2020). *Fusarium* mycotoxins in total mixed rations for dairy cows. Mycotoxin Res..

[51] Gonzalez Pereyra M.L., Chiacchiera S.M., Rosa C.A.d.R., Sager R.L., Dalcero A.M., Cavaglieri L.R. (2012). Fungal and mycotoxin contamination in mixed feeds: Evaluating risk in cattle intensive rearing operations (feedlots). Rev. Bio Cienc..

[52] Yalçin N.F., Işik M.K., Tülay A., Halis O., Çoşkun B., Çiftçi E. (2016). The presence of mycotoxin in total mixed rations of dairy cattle in konya and the surrounding provinces. Atatürk Üniversitesi Vet. Bil. Derg..

[53] Awapak D., Petchkongkaew A., Sulyok M., Krska R. (2021). Co-occurrence and toxicological relevance of secondary metabolites in dairy cow feed from Thailand. Food Addit. Contam. Part A Chem. Anal. Control Expo. Risk Assess..

[54] Custodio L., Prados L.F., Yiannikouris A., Holder V., Pettigrew J., Kuritza L., de Resende F.D., Siqueira G.R. (2019). Mycotoxin contamination of diets for beef cattle finishing in feedlot. R. Bras. Zootec..

[55] Signorini M.L., Gaggiotti M., Molineri A., Chiericatti C.A., de Basilico M.L.Z., Basilico J.C., Pisani M. (2012). Exposure assessment of mycotoxins in cow’s milk in Argentina. Food Chem. Toxicol..

[56] Vaičiulienė G., Bakutis B., Jovaišienė J., Falkauskas R., Gerulis G., Kerzienė S., Baliukonienė V. (2021). Prevalence of Mycotoxins and Endotoxins in Total Mixed Rations and Different Types of Ensiled Forages for Dairy Cows in Lithuania. Toxins.

[57] Kamphues J., Wolf P., Coenen M., Klaus E., Iben C., Kienzle E., Liesegang A., Männer K., Zebeli Q., Zentek J. (2014). IV Beurteilung von den Futtermitteln. Supplemente zur Tierernährung für Studium und Praxis.

[58] Szulc J., Okrasa M., Dybka-Stępień K., Sulyok M., Nowak A., Otlewska A., Szponar B., Majchrzycka K. (2019). Assessment of Microbiological Indoor Air Quality in Cattle Breeding Farms. Aerosol Air Qual. Res..

[59] Hajnal E.J., Kos J., Malachová A., Steiner D., Stranska M., Krska R., Sulyok M. (2020). Mycotoxins in maize harvested in Serbia in the period 2012–2015. Part 2: Non-regulated mycotoxins and other fungal metabolites. Food Chem..

[60] Gruber-Dorninger C., Novak B., Nagl V., Berthiller F. (2017). Emerging mycotoxins: Beyond traditionally determined food contaminants. J. Agric. Food Chem..

[61] Reisinger N., Schurer-Waldheim S., Mayer E., Debevere S., Antonissen G., Sulyok M., Nagl V. (2019). Mycotoxin Occurrence in Maize Silage-A Neglected Risk for Bovine Gut Health?. Toxins.

[62] Gallo A., Ghilardelli F., Atzori A.S., Zara S., Novak B., Faas J., Fancello F. (2021). Co-Occurrence of Regulated and Emerging Mycotoxins in Corn Silage: Relationships with Fermentation Quality and Bacterial Communities. Toxins.

[63] Rychlik M., Humpf H.-U., Marko D., Dänicke S., Mally A., Berthiller F., Klaffke H., Lorenz N. (2014). Proposal of a comprehensive definition of modified and other forms of mycotoxins including “masked” mycotoxins. Mycotoxin Res..

[64] Nesic K., Ivanovic S., Nesic V. (2014). Fusarial toxins: Secondary metabolites of *Fusarium* fungi. Rev. Environ. Contam. Toxicol..

[65] D’Mello J.P.F., Placinta C.M., Macdonald A.M.C. (1999). *Fusarium* mycotoxins: A review of global implications for animal health, welfare and productivity. Anim. Feed Sci. Technol..

[66] Nielsen C., Casteel M., Didier A., Dietrich R., Märtlbauer E. (2009). Trichothecene-induced cytotoxicity on human cell lines. Mycotoxin Res..

[67] Gallo A., Minuti A., Bani P., Bertuzzi T., Cappelli F.P., Doupovec B., Faas J., Schatzmayr D., Trevisi E. (2020). A mycotoxin-deactivating feed additive counteracts the adverse effects of regular levels of *Fusarium* mycotoxins in dairy cows. J. Dairy Sci..

[68] Sy-Cordero A.A., Pearce C.J., Oberlies N.H. (2012). Revisiting the enniatins: A review of their isolation, biosynthesis, structure determination and biological activities. J. Antibiot. Res..

[69] Fink-Gremmels J. (2008). The role of mycotoxins in the health and performance of dairy cows. Vet. J..

[70] Driehuis F., Spanjer M.C., Scholten J.M., Giffel M.C.T. (2008). Occurrence of Mycotoxins in Feedstuffs of Dairy Cows and Estimation of Total Dietary Intakes. J. Dairy Sci..

[71] Driehuis F., Spanjer M.C., Scholten J.M., Te Giffel M.C. (2008). Occurrence of mycotoxins in maize, grass and wheat silage for dairy cattle in the Netherlands. Food Addit. Contam. B Surveill..

[72] Mansfield M.A., Kuldau G.A. (2007). Microbiological and molecular determination of mycobiota in fresh and ensiled maize silage. Mycologia.

[73] Driehuis F. (2013). Silage and the safety and quality of dairy foods: A review. Agric. Food Sci..

[74] Driehuis F., Wilkinson J., Jiang Y., Ogunade I., Adesogan A. (2018). Silage review: Animal and human health risks from silage. J. Dairy Sci..

[75] Magan N., Medina A., Aldred D. (2011). Possible climate-change effects on mycotoxin contamination of food crops pre- and postharvest. Plant Pathol..

[76] Medina Á., Rodríguez A., Magan N. (2015). Climate change and mycotoxigenic fungi: Impacts on mycotoxin production. Curr. Opin. Food Sci..

[77] Medina A., Akbar A., Baazeem A., Rodriguez A., Magan N. (2017). Climate change, food security and mycotoxins: Do we know enough?. Fungal Biol. Rev..

[78] González-Jartín J.M., Rodríguez-Cañás I., Alfonso A., Sainz M.J., Vieytes M.R., Gomes A., Ramos I., Botana L.M. (2021). Multianalyte method for the determination of regulated, emerging, and modified mycotoxins in milk: QuEChERS extraction followed by UHPLC–MS/MS analysis. Food Chem..

[79] Cary J.W., Ehrlich K.C., Bland J.M., Montalbano B.G. (2006). The aflatoxin biosynthesis cluster gene, aflX, encodes an oxidoreductase involved in conversion of versicolorin A to demethylsterigmatocystin. Appl. Environ. Microbiol..

[80] Hsieh D., Lin M., Yao R. (1973). Conversion of sterigmatocystin to aflatoxin B1 by *Aspergillus parasiticus*. Biochem. Biophys. Res. Commun..

[81] Mo H.G., Pietri A., MacDonald S.J., Anagnostopoulos C., Spanjere M. (2015). Survey on sterigmatocystin in food. EFSA Supporting Publ..

[82] Veršilovskis A., de Saeger S. (2010). Sterigmatocystin: Occurrence in foodstuffs and analytical methods—An overview. Mol. Nutr. Food Res..

[83] Parrish F., Wiley B., Simmons E., Long L. (1966). Production of aflatoxins and kojic acid by species of *Aspergillus* and *Penicillium*. Appl. Microbiol..

[84] Morton H.E., Kocholaty W., Junowicz-Kocholaty R., Kelner A. (1945). Toxicity and antibiotic activity of kojic acid produced by *Aspergillus luteo-virescens*. J.Bacteriol..

[85] Kotani T., Ichimoto I., Tatsumi C., Fujita T. (1976). Bacteriostatic activities and metal chelation of kojic acid analogs. Agric. Biol. Chem..

[86] Bashir F., Sultana K., Khalid M., Rabia H. (2021). Kojic Acid: A Comprehensive Review. Asian J. Allied Health Sci..

[87] Ostry V. (2008). *Alternaria* mycotoxins: An overview of chemical characterization, producers, toxicity, analysis and occurrence in foodstuffs. World Mycotoxin J..

[88] Escrivá L., Oueslati S., Font G., Manyes L. (2017). *Alternaria* mycotoxins in food and feed: An overview. J. Food Qual..

[89] EFSA Panel on Contaminants in the Food Chain (2011). Scientific Opinion on the risks for animal and public health related to the presence of *Alternaria* toxins in feed and food. EFSA J..

[90] Aichinger G., Del Favero G., Warth B., Marko D. (2021). *Alternaria* toxins—Still emerging?. Compr. Rev. Food Sci. Food Saf..

[91] Kumari A., Tirkey N.N., Singh K., Srivastava N. (2019). Tenuazonic Acid: A potent mycotoxin. Recent Trends in Human and Animal Mycology.

[92] Gil-Serna J., Vázquez C., Gonzaléz-Jaén M.T., Patiño B., Batt C., Tortorello M.L. (2014). Mycotoxins: Toxicology. Encyclopedia of Food Microbiology.

[93] Schrader T., Cherry W., Soper K., Langlois I., Vijay H. (2001). Examination of *Alternaria alternata* mutagenicity and effects of nitrosylation using the Ames Salmonella test. Teratog. Carcinog. Mutagen..

[94] Aichinger G., Krüger F., Puntscher H., Preindl K., Warth B., Marko D. (2019). Naturally occurring mixtures of *Alternaria* toxins: Anti-estrogenic and genotoxic effects in vitro. Arch. Toxicol..

[95] Vejdovszky K., Warth B., Sulyok M., Marko D. (2016). Non-synergistic cytotoxic effects of *Fusarium* and *Alternaria* toxin combinations in Caco-2 cells. Toxicol. Lett..

[96] Pahlow G., Muck R.E., Driehuis F., Oude Elferink S.J., Spoelstra S.F., Buxton D.R., Muck R.E., Harrison J.H. (2014). Microbiology of ensiling. Silage Science and Technology.

[97] Ogunade I.M., Martinez-Tuppia C., Queiroz O.C.M., Jiang Y., Drouin P., Wu F., Vyas D., Adesogan A.T. (2018). Silage review: Mycotoxins in silage: Occurrence, effects, prevention, and mitigation. J. Dairy Sci..

[98] Wambacq E., Vanhoutte I., Audenaert K., De Gelder L., Haesaert G. (2016). Occurrence, prevention and remediation of toxigenic fungi and mycotoxins in silage: A review. J. Sci. Food Agric..

[99] Malekinejad H., Fink-Gremmels J. (2020). Mycotoxicoses in veterinary medicine: Aspergillosis and penicilliosis. Vet. Res. Forum..

[100] Penagos-Tabares F., Khiaosa-Ard R., Schmidt M., Pacífico C., Faas J., Jenkins T., Nagl V., Sulyok M., Labuda R., Zebeli Q. (2022). Fungal species and mycotoxins in mouldy spots of grass and maize silages in Austria. Mycotoxin Res..

[101] Mansfield M.A., Jones A.D., Kuldau G.A. (2008). Contamination of fresh and ensiled maize by multiple *Penicillium* mycotoxins. Phytopathology.

[102] Oh S.-Y., Boermans H.J., Swamy H.V.L.N., Sharma B.S., Karrow N.A. (2012). Immunotoxicity of *Penicillium* mycotoxins on viability and proliferation of bovine macrophage cell line (BOMACs). Open Microbiol. J..

[103] Brennan K.M., Oh S.-Y., Yiannikouris A., Graugnard D.E., Karrow N.A. (2017). Differential gene expression analysis of bovine macrophages after exposure to the *Penicillium* mycotoxins citrinin and/or ochratoxin a. Toxins.

[104] Oh S.Y., Fisher R.E., Swamy H.V.L.N., Boermans H.J., Yiannikouris A., Karrow N.A., Rios C. (2015). Silage *Penicillium* mycotoxins: Hidden modulators of the immune system. Mycotoxins: Occurrence, Toxicology and Management Strategies.

[105] Nielsen K.F., Sumarah M.W., Frisvad J.C., Miller J.D. (2006). Production of metabolites from the *Penicillium roqueforti* complex. J. Agric. Food Chem..

[106] Malekinejad H., Aghazadeh-Attari J., Rezabakhsh A., Sattari M., Ghasemsoltani-Momtaz B. (2015). Neurotoxicity of mycotoxins produced in vitro by *Penicillium roqueforti* isolated from maize and grass silage. Hum. Exp. Toxicol..

[107] Oh S.Y., Quinton V.M., Boermans H.J., Swamy H.V.L.N., Karrow N.A. (2015). In vitro exposure of *Penicillium* mycotoxins with or without a modified yeast cell wall extract (mYCW) on bovine macrophages (BoMacs). Mycotoxin Res..

[108] Fuchs S., Sontag G., Stidl R., Ehrlich V., Kundi M., Knasmüller S. (2008). Detoxification of patulin and ochratoxin A, two abundant mycotoxins, by lactic acid bacteria. Food Chem. Toxicol..

[109] Oh S.Y., Balch C.G., Cliff R.L., Sharma B.S., Boermans H.J., Swamy H., Quinton V.M., Karrow N.A. (2013). Exposure to *Penicillium* mycotoxins alters gene expression of enzymes involved in the epigenetic regulation of bovine macrophages (BoMacs). Mycotoxin Res..

[110] Bentley R. (2000). Mycophenolic acid: A one hundred year odyssey from antibiotic to immunosuppressant. Chem. Rev..

[111] Kopp-Holtwiesche B., Rehm H. (1990). Antimicrobial action of roquefortine. J. Environ. Pathol. Toxicol..

[112] Noto T., Sawada M., Ando K., Koyama K. (1969). Some biological properties of mycophenolic acid. J. Antibiot. Res..

[113] Lu X., Zhang E., Yin S., Fan L., Hu H. (2017). Methylseleninic acid prevents patulin-induced hepatotoxicity and nephrotoxicity via the inhibition of oxidative stress and inactivation of p53 and MAPKs. J. Agric. Food Chem..

[114] Zhang B., Huang C., Lu Q., Liang H., Li J., Xu D. (2022). Involvement of caspase in patulin-induced hepatotoxicity in vitro and in vivo. Toxicon.

[115] Manni K., Rämö S., Franco M., Rinne M., Huuskonen A. (2022). Occurrence of Mycotoxins in Grass and Whole-Crop Cereal Silages—A Farm Survey. Agriculture.

[116] Auerbach H., Oldenburg E., Weissbach F. (1998). Incidence of *Penicillium roqueforti* and roquefortine C in silages. J. Sci. Food Agric..

[117] Magan N., Lacey J. (1984). Effect of water activity, temperature and substrate on interactions between field and storage fungi. Trans. Brit. Mycol. Soc..

[118] Marín S., Sanchis V., Sáenz R., Ramos A., Vinas I., Magan N. (1998). Ecological determinants for germination and growth of some *Aspergillus* and *Penicillium* spp. from maize grain. J. Appl. Microbiol..

[119] Mislivec P., Dieter C., Bruce V. (1975). Effect of temperature and relative humidity on spore germination of mycotoxic species of *Aspergillus* and *Penicillium*. Mycologia.

[120] Skladanka J., Adam V., Dolezal P., Nedelnik J., Kizek R., Linduskova H., Mejia J.E.A., Nawrath A. (2013). How do grass species, season and ensiling influence mycotoxin content in forage?. Int. J. Environ. Res..

[121] Woolford M.K. (1990). The detrimental effects of air on silage. J. Appl. Bacteriol..

[122] Ulrich S., Gottschalk C., Biermaier B., Bahlinger E., Twarużek M., Asmussen S., Schollenberger M., Valenta H., Ebel F., Dänicke S. (2021). Occurrence of type A, B and D trichothecenes, zearalenone and stachybotrylactam in straw. Arch. Anim. Nutr..

[123] Bhat R., Rai R.V., Karim A.A. (2010). Mycotoxins in food and feed: Present status and future concerns. Compr. Rev. Food Sci. Food Saf..

[124] Nasr H., Pearson O. (1975). Inhibition of prolactin secretion by ergot alkaloids. Eur. J. Endocrinol..

[125] Coufal-Majewski S., Stanford K., McAllister T., Blakley B., McKinnon J., Chaves A.V., Wang Y. (2016). Impacts of cereal ergot in food animal production. Front. Vet. Sci..

[126] Bauer J.I., Gross M., Cramer B., Humpf H.-U., Hamscher G., Usleber E. (2018). Immunochemical analysis of paxilline and ergot alkaloid mycotoxins in grass seeds and plants. J. Agric. Food Chem..

[127] Canty M.J., Fogarty U., Sheridan M.K., Ensley S.M., Schrunk D.E., More S.J. (2014). Ergot alkaloid intoxication in perennial ryegrass (*Lolium perenne*): An emerging animal health concern in Ireland?. Ir. Vet. J..

[128] Evans T.J. (2011). Diminished reproductive performance and selected toxicants in forages and grains. Vet. Clin. N. Am. Food Anim. Pract..

[129] Marczuk J., Zietek J., Zwierz K., Winiarczyk S., Lutnicki K., Brodzki P., Adaszek L. (2019). Ergovaline poisoning in a herd of dairy cows—A case report. Med. Weter..

[130] Miethbauer S., Gaube F., Möllmann U., Dahse H.-M., Schmidtke M., Gareis M., Pickhardt M., Liebermann B. (2009). Antimicrobial, antiproliferative, cytotoxic, and tau inhibitory activity of rubellins and caeruleoramularin produced by the phytopathogenic fungus *Ramularia collo-cygni*. Planta Med..

[131] Walters D.R., Havis N.D., Oxley S.J. (2008). Ramularia collo-cygni: The biology of an emerging pathogen of barley. FEMS Microbiol. Lett..

[132] Hayakawa S., Minato H., Katagiri K. (1971). The ilicicolins, antibiotics from *Cylindrocladium ilicicola*. J. Antibiot. Res..

[133] Aldridge D., Turner W. (1970). Metabolites of *Helminthosporium monoceras*: Structures of monocerin and related benzopyrans. J. Chem. Soc. C..

[134] Robeson D., Strobel G. (1982). Monocerin, a phytotoxin from *Exserohilum turcicum* (≡ *Drechslera turcica*). Agric. Biol. Chem..

[135] Jouda J.-B., Tamokou J.-d.-D., Mbazoa C.D., Douala-Meli C., Sarkar P., Bag P.K., Wandji J. (2016). Antibacterial and cytotoxic cytochalasins from the endophytic fungus *Phomopsis* sp. harbored in Garcinia kola (Heckel) nut. BMC complement. Altern. Med..

[136] Aldridge D., Armstrong J., Speake R., Turner W. (1967). The cytochalasins, a new class of biologically active mould metabolites. Chem. Commun..

[137] Oh M., Son H., Choi G.J., Lee C., Kim J.C., Kim H., Lee Y.W. (2016). Transcription factor ART 1 mediates starch hydrolysis and mycotoxin production in *Fusarium graminearum* and *F. verticillioides*. Mol. Plant Pathol..

[138] Rotem J. (1994). The Genus Alternaria: Biology, Epidemiology, and Pathogenicity.

[139] Drakopoulos D., Sulyok M., Krska R., Logrieco A.F., Vogelgsang S. (2021). Raised concerns about the safety of barley grains and straw: A Swiss survey reveals a high diversity of mycotoxins and other fungal metabolites. Food Control.

[140] Coop I.E. (1977). Depression of lambing performance from mating on lucerne. Proc. N. Z. Soc. Anim. Prod..

[141] Mostrom M., Evans T.J., Gupta R.C. (2011). Phytoestrogens. Reproductive and Developmental Toxicology.

[142] Wyse J.M., Latif S., Gurusinghe S., Berntsen E.D., Weston L.A., Stephen C.P. (2021). Characterization of Phytoestrogens in *Medicago sativa* L. and Grazing Beef Cattle. Metabolites.

[143] EFSA (2007). Opinion of the scientific panel on contaminations in the food chain on a request from the commission related to cyanogenic compounds as undesirable substances in animal feed. EFSA J..

[144] Vetter J. (2000). Plant cyanogenic glycosides. Toxicon.

[145] Gurnsey M., Jones W., Merrall M., Reid C. (1977). Cyanide poisoning in cattle: Two unusual cases. N. Z. Vet. J..

[146] Dong X., Fu J., Yin X., Cao S., Li X., Lin L., Huyiligeqi, Ni J. (2016). Emodin: A review of its pharmacology, toxicity and pharmacokinetics. Phytother. Res..

[147] Kiyoshi K., Taketoshi K., Hideki M., Jiro K., Yoshinori N. (1984). A comparative study on cytotoxicities and biochemical properties of anthraquinone mycotoxins emodin and skyrin from *Penicillium islandicum* Sopp. Toxicol. Lett..

[148] Anderson R.C., Majak W., Rassmussen M.A., Callaway T.R., Beier R.C., Nisbet D.J., Allison M.J. (2005). Toxicity and metabolism of the conjugates of 3-nitropropanol and 3-nitropropionic acid in forages poisonous to livestock. J. Agric. Food Chem..

[149] Ludolph A., He F., Spencer P., Hammerstad J., Sabri M. (1991). 3-Nitropropionic acid-exogenous animal neurotoxin and possible human striatal toxin. Can. J. Neurol. Sci..

[150] Maiya S., Grundmann A., Li S.M., Turner G. (2006). The fumitremorgin gene cluster of *Aspergillus fumigatus*: Identification of a gene encoding brevianamide F synthetase. Chembiochem.

[151] Rahman A., Siddiqui S.A., Rahman M.O., Kang S.C. (2020). Cyclo (L-Pro-L-Tyr) from *Streptomyces* sp. 150: Exploiting in vitro Potential in Controlling Foodborne Pathogens and Phytopathogens. Antiinfect. Agents.

[152] Zin N.M., Al-Shaibani M.M., Jalil J., Sukri A., Al-Maleki A.R., Sidik N.M. (2020). Profiling of gene expression in methicillin-resistant Staphylococcus aureus in response to cyclo-(l-Val-l-Pro) and chloramphenicol isolated from *Streptomyces* sp., SUK 25 reveals gene downregulation in multiple biological targets. Arch. Microbiol..

[153] VDLUFA (2012). Die Chemische Untersuchung von Futtermitteln. Handbuch der Landwirtschaftlichen Versuchs- und Untersuchungsmethodik (VDLUFA-Methodenbuch).

[154] Van Soest P.V., Robertson J.B., Lewis B. (1991). Methods for dietary fiber, neutral detergent fiber, and nonstarch polysaccharides in relation to animal nutrition. J. Dairy Sci..

[155] Lammers B., Buckmaster D., Heinrichs A. (1996). A simple method for the analysis of particle sizes of forage and total mixed rations. J. Dairy Sci..

[156] Kononoff P., Heinrichs A., Buckmaster D. (2003). Modification of the Penn State forage and total mixed ration particle separator and the effects of moisture content on its measurements. J. Dairy Sci..

[157] Sulyok M., Stadler D., Steiner D., Krska R. (2020). Validation of an LC-MS/MS-based dilute-and-shoot approach for the quantification of >500 mycotoxins and other secondary metabolites in food crops: Challenges and solutions. Anal. Bioanal. Chem. Res..

[158] Steiner D., Sulyok M., Malachová A., Mueller A., Krska R. (2020). Realizing the simultaneous liquid chromatography-tandem mass spectrometry based quantification of >1200 biotoxins, pesticides and veterinary drugs in complex feed. J. Chormatogr. A..

[159] Shimshoni J.A., Cuneah O., Sulyok M., Krska R., Galon N., Sharir B., Shlosberg A. (2013). Mycotoxins in corn and wheat silage in Israel. Food Addit. Contam. Part A Chem. Anal. Control Expo. Risk Assess..

[160] Nichea M.J., Palacios S.A., Chiacchiera S.M., Sulyok M., Krska R., Chulze S.N., Torres A.M., Ramirez M.L. (2015). Presence of multiple mycotoxins and other fungal metabolites in native grasses from a wetland ecosystem in Argentina intended for grazing cattle. Toxins.

[161] Hinkle D.E., Wiersma W., Jurs S.G. (2003). Correlation. Applied Statistics for the Behavioral Sciences.

[162] Oldick B., Firkins J., St-Pierre N. (1999). Estimation of microbial nitrogen flow to the duodenum of cattle based on dry matter intake and diet composition. J. Dairy Sci..

