# Supplementary material for: Cocktails of Mycotoxins, Phytoestrogens, and Other Secondary Metabolites in Diets of Dairy Cows in Austria: Inferences from Diet Composition and Geo-Climatic Factors
Source: Toxins (Basel). 2022 Jul 15;14(7):493. doi: 10.3390/toxins14070493 (PMC9318294; doi:10.3390/toxins14070493)

Cocktails of Mycotoxins, Phytoestrogens, and Other Secondary Metabolites in Diets of Dairy Cows in Austria: Inferences from Diet Composition and Geo-climatic Factors

Felipe Penagos-Tabares, Ratchaneewan Khiaosa-ard, Marlene Schmidt, Eva-Maria Bartl, Johanna Kehrer, Veronika Nagl, Johannes Faas, Michael Sulyok, Rudolf Krska and Qendrim Zebeli

Table S1. List of 863 targeted metabolites via a validated multi-metabolite liquid chromatography/electrospray ionization-tandem mass spectrometric (LC/ESI–MS/MS). * Compounds found in diets of Austrian dairy cows (values > the LOD)

10-Norparvulenone	Aflatoxin M2	Apicidin	Aurantine
15-Acetyldeoxynivalenol	Aflatoxin P1	Apidicin C	Aurantioclavin
15-Desoxyoxalicine B	Aflatoxin Q1	Apidicin D2*	Aurantiogliocladin
15-Hydroxyculmorin*	Aflatrem	Aristolochic acid A	Aurasperon B
15-Hydroxyculmoron	Aflavarin	Ascochlorin*	Aurasperon C
16-Ketoaspergillimide	Agistatin B	Ascofuranone*	Aurasperon G
1-Deoxypebrolide	Agistatin D	Ascolactone	Aureobasidin
2-Chlorunguinol	Agistatin E	Ascomycin	Aurofusarin*
2-Methylmitorubin	Agroclavine	Asparason A	Austalide A
3,4,15Triacetylnivalenol	Aigualomycin D	Aspercolorin	Austalide B
3,4-Diacetylnivalenol	AJ 296	Asperflavine	Austalide Derivative
3-Acetyldeoxynivalenol	Alamethicin*	Asperfuran	Austalide F
3-Acetylneosolaniol	alpha-Zearalenol	Aspergamid A	Austamide
3-Acetyl-T-2 Toxin	alpha-Zearalenol Glucoside	Aspergillicin Derivat	Austdiol
3-Hydroxy-3-acetyl-T-2 Toxin	Alteichin	Aspergillimide	Austinol
3-Hydroxy-HT-2 Toxin	Altenuene	Asperglaucide*	Austocystin A
3-Hydroxyterphenyllin	Altenuisol*	Asperlactone	Austocystin B
3-Nitropropionic acid*	Altenusin	Asperloxine A	Austocystin D
4,7,15Triacetylnivalenol	Alternarian acid	Aspermytin A	Austocystin I
4-Hydroxyalternariol	Alternarienoic acid	Aspernigrin A	Australide D
4-Methoxycyclopeptin	Alternariol*	Asperphenamate*	Australide F
4-Monoacetoxyscirpenol	Alternariol-3-Glucoside	Asperthecin	Averantin
5-Hydroxyculmorin	Alternariol-9-Glucoside	Aspinolid B	Averantinmethylether
5-Methylmellein	Alternariolmethylether*	Aspinonene	Averufanin
7-Hydroxykaurenolide	Alternariolmethylether-Glucoside	Aspochalasin C	Averufin Derivat
7-Hydroxypestalotin*	Altersetin*	Aspochalasin D	Averufin*
8-Acetylneosolaniol	Altersolanol	Aspochalasin H	Bacitracin
8-O-Methylaverufin	Altertoxin II	Aspochalasin I	Bafilomycin A1
A 23187	Altertoxin-I	Aspochalasin J	Banksialactone A
A 26771 B	Amauromine	Aspochracin	Barceloneic acid*
AAL TA-Toxin	Amidepsin B	Aspterric acid	Bassianolide*
AAL TB Toxin	Aminodimethyloctadecanol	Aspulvinone E	Beauvericin*
AAL TD Toxin	Amoxycillin	Aspulvinone O	Benzomalvin A
AAL TE Toxin	Amphotericin	Aspyrone	Benzomalvin B
Abscisic acid*	Anacin	Asteltoxin	Benzomalvin C
Acetylchaetoglobosin D	Andrastin A*	Asterric acid	Berkedrimane B
Achaetolide Derivat	Andrastin B*	Asterriquinonedimethylether	Berkeleyacetal B
Acuminatum B*	Andrastin C*	Aszonapyrone A	Berkeleylctone E
Acuminatum C	Andrastin D	Atlantinon A	Berkeleylctone F
Aflatoxicol	Andrastin Derivative	Atpenin A5	beta-Zearalenol
Aflatoxin B1	Anisomycin	Atropine	beta-Zearalenol-Glucoside
Aflatoxin B2	Antbiotic L 696474	Atroventinmethylether	Bikaverin*
Aflatoxin G1	Antibiotic F 1849 A	Aurantiamin A	Biochanin*
Aflatoxin G2	Antibiotic PF 1052	Auranticin A	Bis(methylthio)gliotoxin*
Aflatoxin M1	Antibiotic Y*	Auranticin A	Bongkrekic acid

Botryodiplodin	cyclo(L-Pro-L-Val)*	Dihydrolysergol	Fonsecin
Brasiliamide A	Cycloaspeptide A	Dihydrosterigmatocystin	Formonetin*
Brefeldin A	Cycloechinulin	Dihydrotrichotetronine	FS-4
Brevianamid F*	Cycloheximide	Dihydroxycalonectrin	Fulvic acid
Brevicompanine B	Cyclopenin	Dihydroxymellein	Fumagillin
Butenolid	Cyclopenol	DihydroxyZONMethylether	Fumarprotocetaric acid
Butyrolacton III	Cyclopeptine	Dinactin	Fumifungin
Butyrolactone I	Cyclopiazonsäure	Diplodiatoxin	Fumigaclavine C*
Butyrolactone II	Cyclosporin A	DON-3-glucoside*	Fumigaclavine*
ButyrolactonIImethylether	Cyclosporin B*	Doxorubicin	Fumiquinazolin A
Byssochlamic acid	Cyclosporin C*	Doxycyclin	Fumiquinazolin D*
Calonectrin	Cyclosporin D	Drimane 6	Fumiquinazolin Derivat
Calphostin	Cyclosporin H	Drimane 8	Fumiquinazolin F
Calphostin C*	Cylindrocarpon A4	Duclauxin	Fumitremorgin A
Calyxanthone	Cylindrol B	Echimidin	Fumitremorgin B
Carnequinazolin A	Cytochalasin A	Elymoclavine	Fumitremorgin C
Cephalochromin	Cytochalasin B	Elymoclavine-Fructoside	Fumonisin A1
Cercosporamide	Cytochalasin C	Emericellamide A	Fumonisin A1 (precussor)*
Cercosporin	Cytochalasin D	Emericellamide C	Fumonisin A2
Cereulide	Cytochalasin E	Emericellamide E	Fumonisin AK2
Cerulenin	Cytochalasin J	Emestrin*	Fumonisin B1*
Chaconin*	Daidzein*	Emindole SA	Fumonisin B2*
Chaetocin	Daidzin*	Emodin*	Fumonisin B3*
Chaetoglobosin A	Daunorubicin	Endocrocin*	Fumonisin B4*
Chaetoglobosin C	Deacetylneosolaniol	Enniatin A*	Fumonisin B6
Chaetoglobosin D	Decalonectrin	Enniatin A1*	Fungerin
Chaetoglobosin F	Dechlorogriseofulvin	Enniatin B*	Fusaproliferin*
Chaetominine	Dechlorogriseofulvin	Enniatin B1*	Fusapyron*
Chaetoviridin A	Dechloroisochromophilon IV	Enniatin B2*	Fusarenon-X
Chanoclavin*	Dechloronornidulin	Enniatin B3	Fusaric acid
Chetomin	Deepoxy-deoxynivalenol	Epiequisetin*	Fusarielin A
Chetoseminudin A	Deepoxy-T-2 toxin	Epoxyagroclavin	Fusarin C
Chevalone B	Deepoxy-T-2tetraol	Epoxycytochalsin C*	Fusarinolic acid
Chevalone C	Dehydroaustinol	Equisetin*	Fusarisetin A
Chevalone E	Dehydrocurvularin*	Eremofortin A	Fuscofusarin
Chlamydospordiol	Dehydrocyclopeptine	Eremofortin B	Galbinic acid
Chlamydosporol	Dehydrogriseofulvin	Ergine	Geldanamycin
Chloramphenicol	Demethoxyviridol	Ergocornine*	Genistein*
Chlorocitreorosein	Demethylasteltoxin	Ergocorninin*	Genistin*
Chloronectrin	Demethylsulochrin	Ergocristine*	Geodin
Chlortetracyclin	DeoxyAltersolanol	Ergocristinine*	Geodin hydrate
Chrodrimanin	Deoxybrevianamid E	Ergocryptine*	Gibberellic acid
Chromomycin A3	Deoxyfusapyron	Ergocryptinine*	Gibberellin A12
Chrysogin*	Deoxygerfelin*	Ergometrine	Gibberellin A14
Chrysophanol*	Deoxynivalenol*	Ergometrinine*	Gibberellin A4
Cinereanin	Deoxynortryptoquivalin*	Ergosin*	Gibberellin A7
Citreohybriddione	Deoxytryptoquialanine	Ergosinin*	Gibepyron D
Citreohybridinol*	Deoxytryptoquivaline A	Ergotamine*	Gigantenone
Citreoindole	Desoxypaxillin	Ergotaminine*	Gliocladic acid
Citreorosein*	Desoxyverrucosidin	Ergovalin	Gliotoxin
Citreoviridin	Destruxin A	Erucifolin	Glisoprenin D
Citreoviridin C	Destruxin B*	Erucifolin-N-Oxid	Glyantrypine
Citreoviridinol	Destruxin CHL	Erythromycin	Glycitein*
Citrinin*	Destruxin D	Ethylorsellinic acid	Glycitin*
Citromycetin	Destruxin-Ed Derivat	Europin	Grayanotoxin I
Cladosporin	Dethiosecoemestrin	Europin-N-Oxid	Griseofulvin acid
Cladosporone Derivat	Diacetoxyscirpenol	Expansolid	Griseofulvin*
Clonostachydiol	Diacetylcercosporin	F01 1358-A	Griseophenone A
CNM 115443	Diacetylnivalenol	Fallacinol	Griseophenone B
Cochlioquinone A	Dichlordiaportin	FB1 Methylester	Griseophenone C
Colchicine*	Dichlormethylasterric acid	Fellutanine A*	Harzianopyridine
Communesin B	Diffractaic acid	Fellutannine B	Harzianum A
Cordycepin	Dihydroaspyrone	Festuclavine*	HC Toxin
Coumestrol*	Dihydrochlamydocin	Filipin	Heliotrin
Culmorin*	Dihydrocitrinone	FK 506	Heliotrin-N-Oxid
Curvularin*	Dihydrocompactin	FK 9775 A	Helvolic acid
Curvulin	Dihydroergosine	FK 9775 B	Helvolinic acid
Cyclo(l-Ala-L-Pro)	Dihydroergotamine	Flavipucin	Heptaibin
cyclo(L-Leu-L-Pro)	Dihydrogriseofulvin	Flavoglaucin*	Heptelidic acid
cyclo(L-Pro-L-Tyr)*	Dihydroinfectopyron	Folipastin	Herquline A

Hexaacetyl-HFB1	Lysergol	Ochrephilone	Pyrenocin A
Hirsutide	Macrosphelide A	Okaramine B	Pyrenophorol*
HT-2 Glucoside*	Macrosporin	Okaramine D	Pyripyropene A
HT-2 toxin*	Malformin A	Oligomycin A	Pyripyropene B
hydrolysed Fumonisin B1	Malformin A2	Oligomycin B	Pyripyropene D
hydrolysed Nidulin	Malformin C	O-Methylsterigmatocystin	Pyrophen
Hydroxyandrastin A	Marcfortine A*	O-Methylviridicatin	Quadrone
Hydroxyandrastin C*	Marcfortine C*	Ononin*	Questiomycine A*
Hydroxycarnequinazolin A	Meleagrin	Ophiobolin A	Questiomycine Derivat*
Hydroxycurvularin	Meleagrin Derivative	Ophiobolin B	Questiomycine*
Hydroxypaspaline	MER-NF5003E	Orsellinic acid	Quinadoline A
Hydroxysulchorin	MER-NF5003F	Oxalicine B	Quinadoline B
Hydroxysydonic acid	Methoxycurvularin	Oxaline	Quinolactacin A
Hyoscin	Methoxysterigmatocystin	Oxidized Elymoclavine	Quinolactacin B
Hypothemycin	Methylasterric acid	Oxidized Luol	Quinolone A
Ilicicolin A*	Methylequisetin	Oxisterigmatocystin E	Radicicol
Ilicicolin B*	Methylfunicone	Oxyskyrin*	Radicinin*
Ilicicolin C	Methylorsellinic acid	Papyracillic acid A	Radicinol
Ilicicolin E*	Methylsulochrin*	Paracelsin A	Radiclonic acid
Ilicicolin F	Mevastatin	Paracelsin B	Rapamycin
Ilicicolin H*	Mevinolin*	Paraherquamide A	Rasfonin
Indicin_IM_LA	Mithramycin C	Paraherquamide E	Retrorsin
Indicin_IM_LA-N-Oxid	Mitomycin	Paspalic acid	Roquefortine C*
Infectopyron*	Mitorubinic acid	Paspalin	Roquefortine D*
Integracin A*	Mollicellin D	Paspalinin	Roquefortine E
Integracin B*	Monactin	Paspalitrem A	Roridin A
Ionomycin	Moniliformin*	Paspalitrem B	Roridin L2
Irgasan	Monoacetoxyscirpenol*	Patulin	Roritoxin C
Isocereulide A	Monocerin*	Paxillin	Rorotoxin A
Isochromophilon III	Monocrotalin	Pencillazaphilone B	Rosellichalasin
Isochromophilon IV	Monocrotalin-N-Oxid	Penicillic acid	Rubellin D*
Isochromophilon IX	Monomethylcurvulin	Penicillide	Rubratoxin A
Isochromophilone VI	MPA Derivative	Penicillin G	Rubrofusarin
Isofusidienol	Mycophenolic acid IV	Penicillin V	Rugulosin
Isokotanin B	Mycophenolic acid*	Penicinoline	Rugulotrosin
Isopenicillide Derivative	Myriocin*	Penicolinate	Rugulovasine A
Iso-Rhodoptilometrin*	Mytoxin C	Penigequinolone A	Rugulusovine*
Isosulochrin	N-0352A	Peniprequinolone	Salazinic acid
Jacobin	N-0532B	Penitrem A	Sambucinol
Jacobin-N-Oxid	N-Acetyl-HFB1	Pennigritrem A	Sartorypron
Janthitrem A	N-Benzoyl-Phenylalanine*	Pentahydroxyscirpenol	Sartorypyrone B
Josamycin	Neoechinulin A*	Pentoxyfylline	Satratoxin F
K252a	Neosartorin	Pestalone	Satratoxin G
K252b	Neosolaniol	Pestalotin*	Satratoxin H
K-76 Derivative 4	Neoxaline	Petromurin C	Scalusamid A
Kipukasin A	NG 012	PF 1163 A	Sch 725680
Kipukasin B	Nidulin	Phaseolinone	Scirpentriol
Kipukasin D	Nidurufin	Phenopyrrozin*	Sclerotigenin
KO 143	Nigericin	Phenylpyropene A	Sclerotin A
Kojic acid*	Nigragillin	Phomalone	Sclerotioramin
Koninginin A	Nigrosporoate A	Phomopsidin	Sclerotiorin
Koninginin B	Nivalenol Glucoside	Phomopsin A	Secalonic acid B
Koninginin D	Nivalenol*	Phomopsolide B	Secalonic acid D
Koninginin E	Nocardamine	Phomoxanthone A	Secalonic acid F
Kotanin A	Nonactin	Phthalexin	Secoemestrin C Derivat
Kumbicin C	Norcitreoviridin	p-Hydroxyphenopyrrozin	seco-Sterigmatocystin
Lasiocarpin	Norlichexanthone*	Physcion*	semi Xanthomegnin
Lasiocarpin-N-Oxid	Nornidulin	Pinselin	semi-Vioxanthin
Lecanoric acid*	Norsolorinic acid	Piscarinin A	Senecionin_Senecivernin
Leoidin	Norstictic acid	Porritoxinol	Senecionin_Senecivernin_N-Oxid
Linamarin*	Nortryptoquialanine	Prehelminthosporol	Seneciphylline
Lincomycin	Norverrucosidin	Prehelminthosporollacton	Seneciphylline-N-Oxide
LL-Z 1272e*	Notoamide Derivative	Prelaptin	Senkirkin
LLZ 1640-2	Notoamide E Derivat	Preussin	Setusosin
LLZ 1640-4	NT-2 Toxin	Pseurotin A	Siccanin
Lolitrem B	Obscurolide A1	Puberulin A	Siccanol*
Lolitrem N	Ochratoxin A*	Puromycin	Sissotrine
Lotaustralin*	Ochratoxin alpha	Purpactin A	Skyrin*
Luteoskyrin	Ochratoxin B	Purpuride	S-MethylDON
Luteusin A	Ochratoxin C	Pyranonigrin	Sorbicillacton A

Sphingofungin B	Tenuazonic acid*	Trichoverrin A	Versiconol
Sphingofungin D	Ternatin*	Trypacidin*	Verticillin A
Spiculisporic acid	Terpendole C	Tryprostatin A	Violaceic acid
Spiramycin	Terpendole E	Tryprostatin B	Violaceol I
Spirodihydrobenzofuranlactam IV	Terpendole I	Tryptophol*	Violaceol II
Sporidesmolide II*	Terphenyllin	Tryptoquialanine	Viomellein
Sporogen AO I	Terragine E	Tryptoquialanine Derivat	Vioxanthin
Stachybotryamide	Terrecyclic acid	Tryptoquivaline A	Viridicatin
Stachybotrylactam	Terrein	Tryptoquivaline F	Viridicatol
Stachybotrysin B	Terretonin	Tryptoquivaline G	Viridicatum toxin
Staurosporin	Terretonin F Derivat	Tylosin	Viriditoxin
Stemphylperylenol	Territrem A	Ulocladol	Viridol
Sterigmatocystin*	Territrem B	Unguinol	Vulpinic acid
Sticitc acid	Tetraacetlnivalenol	Unugisin E	W493*
Strobilactone A	Tetraacetyl-T-2 Tetraol	Usnic acid*	WIN 68577
Sulochrin	Tetracycline	Ustiloxin A	WIN-64821
Surfactin A	Tetrahydrobostrycin	Ustiloxin B	Wortmannin
Surfactin B	Thailandolide B	Ustiloxin D	Xanthomegnin
Sydonic acid	Thaxtomin A	Ustusol A	Xanthotoxin*
Sydonol	Thielavin B	Valinomycin	Xantocillin X1
Sydowinin A	Toxoflavin	Vancomycin	Yaequinolone J2
Sydowinin B	Triacetoxyscirpenol	Vermistatin	Zearalenone*
sydowinol	Triacetyl-Deoxynivalenol	Verrucarin A	Zearalenone-14-glucoside
T-2 Glucoside	Trichalasin B	Verrucarin J	Zeralenone-16-Glucoside
T-2 toxin*	Trichodermamide C	Verrucarol	Zinndiol*
T2-Tetraol	Trichodermin	Verrucofortine	Zinniamide
T2-Triol	Trichodesmin	Verrucosidin	Zinniol*
Tanzawaic acid B	Trichodimerol	Verruculogen	
Taxol	Trichostatin A	Verruculotoxin	
Tenellin	Trichotetronine	Versicolorin A	
Tensidol B	Trichothecin	Versicolorin C*	
Tentoxin*	Trichothecolone	Versiconal Acetat (Hemiacetal)	

Table S2. Odds ratio estimates and profile-likelihood confidence intervals of forage inclusion levels as dietary risk factors for high *Fusarium* mycotoxin loads (above 75th percentile concentrations)

**Forage sources**	**Estimate**	**95% confidential limits**	
Maize silage	1.085	1.007	1.168
Grass silage	0.966	0.908	1.023
Straw	2.004	1.372	3.193
Hay	0.997	0.850	1.215
Brewer spent grain silage	1.056	0.792	1.446
Other silage	1.624	1.086	2.689

Figure S1. Predicted probabilities for high *Fusarium* mycotoxin loads (above 75th percentile concentrations) related to the proportion of (**A**) maize silage and (**B**) straw in the dietary rations of Austrian dairy cows.

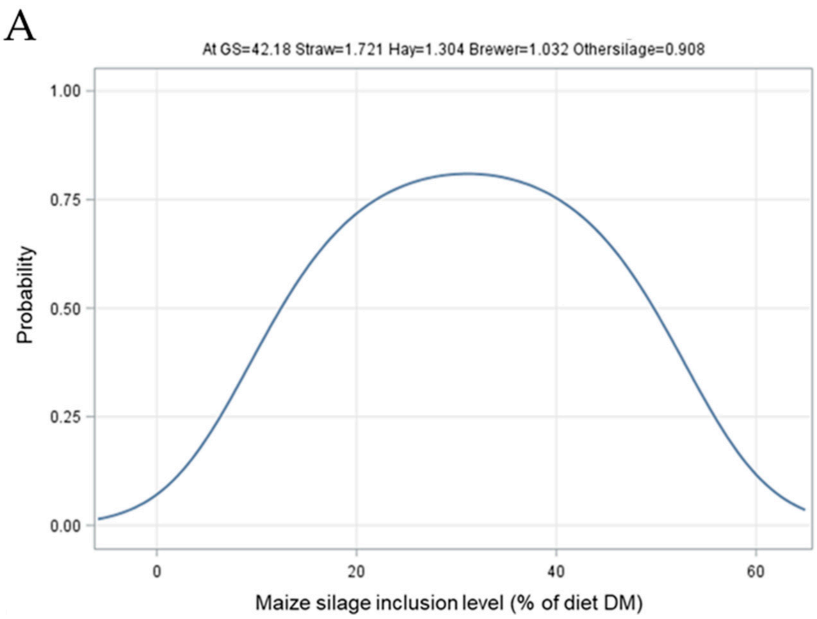

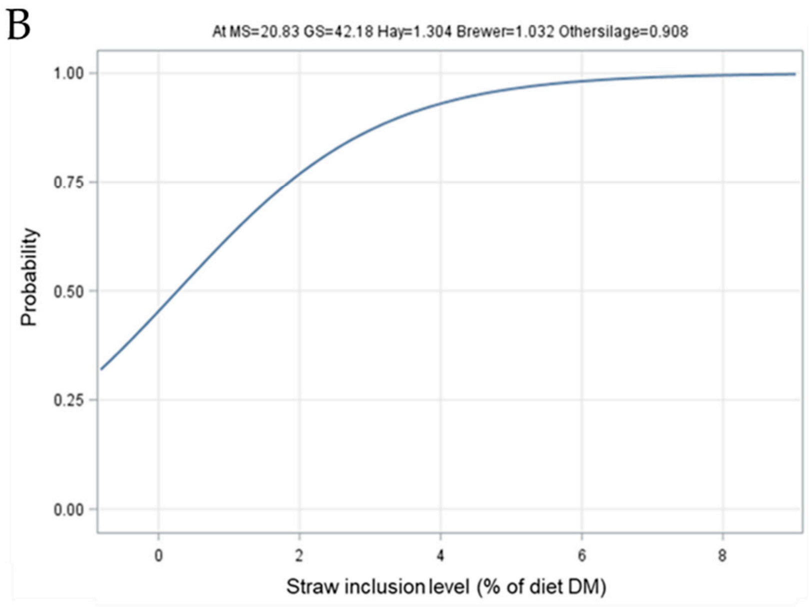

Supplement: Supplementary file 1 [file toxins-14-00493-s001.zip › toxins-1787161-supplementary.pdf]
